# Preclinical and clinical activity of DZD1516, a full blood–brain barrier-penetrant, highly selective HER2 inhibitor

**DOI:** 10.1186/s13058-023-01679-4

**Published:** 2023-07-06

**Authors:** Jian Zhang, Nicholas P. McAndrew, Xiaojia Wang, Yiqun Du, Brian DiCarlo, Mei Wang, Kan Chen, Wenlei Yu, Xichun Hu

**Affiliations:** 1 Phase I Clinical Trial Center, Fudan University Shanghai Cancer Center, Shanghai, People’s Republic of China; 2 Department of Oncology, Shanghai Medical College, Fudan University, Shanghai, People’s Republic of China; 3 Division of Hematology/Oncology, UCLA David Geffen School of Medicine, Los Angeles, CA USA; 4 Zhejiang Cancer Hospital, Zhejiang, People’s Republic of China; 5 Dizal Pharmaceutical, Shanghai, People’s Republic of China; 6 Department of Medical Oncology, Fudan University Shanghai Cancer Center, Shanghai, 200032 People’s Republic of China

**Keywords:** HER2, Tyrosine kinase inhibitor, Blood–brain barrier, Breast cancer, Central nervous system metastases

## Abstract

**Background:**

Patients with HER2-positive metastatic breast cancer (MBC) are at high risk of developing central nervous system (CNS) metastases. A potent and selective HER2 inhibitor with good blood–brain barrier (BBB) penetration is highly desirable.

**Methods:**

The design and structure–activity relationship of DZD1516 was described. The potency and selectivity of DZD1516 were determined by enzymatic and cellular assays. The antitumor activity of DZD1516 monotherapy or in combination with HER2 antibody–drug conjugate was assessed in CNS and subcutaneous xenograft mouse models. A phase 1 first-in-human study evaluated the safety, tolerability, pharmacokinetics, and preliminary antitumor activity of DZD1516 in patients with HER2+ MBC who relapsed from standard of care.

**Results:**

DZD1516 showed good selectivity against HER2 over wild-type EGFR in vitro and potent antitumor activity in vivo. Twenty-three patients were enrolled and received DZD1516 monotherapy treatment across six dose levels (25–300 mg, twice daily). Dose-limiting toxicities were reported at 300 mg, and thus 250 mg was defined as the maximum tolerated dose. The most common adverse events included headache, vomiting, and hemoglobin decreased. No diarrhea or skin rash was observed at ≤ 250 mg. The mean *K*_p,uu,CSF_ was 2.1 for DZD1516 and 0.76 for its active metabolite DZ2678. With median seven lines of prior systemic therapy, the best antitumor efficacy in intracranial, extracranial and overall lesions was stable disease.

**Conclusions:**

DZD1516 provides positive proof of concept for an optimal HER2 inhibitor with high BBB penetration and HER2 selectivity. Further clinical evaluation of DZD1516 is warranted, with the RP2D being 250 mg BID.

*Clinicaltrials.gov identifier* NCT04509596. Registered on August 12, 2020; Chinadrugtrial: CTR20202424 Registered on December 18, 2020.

**Supplementary Information:**

The online version contains supplementary material available at 10.1186/s13058-023-01679-4.

## Background

Breast cancer is one of the most common cancers worldwide [1]. About 15% ~ 30% of breast cancer overexpresses human epidermal growth factor receptor 2 (HER2) [2]. Current FDA-approved drugs for HER2-positive (HER2 +) metastatic breast cancer (MBC) include antibodies (such as trastuzumab and pertuzumab) [3], antibody–drug conjugates (ADCs, such as T-DM1 and T-DXd) [4, 5], and tyrosine kinase inhibitors (TKIs, such as lapatinib, neratinib, and tucatinib) in combination with chemotherapy [6–8].

Although these drugs have been proven effective in treating HER2+ breast cancer, there is still an unmet medical need for patients who relapsed from or were refractory to these therapies. For patients with central nervous system (CNS) metastases, including brain metastasis (BM) and leptomeningeal metastasis (LM), their prognosis was even worse [9, 10]. Up to 50% of patients relapsed with CNS metastases while receiving HER2-targeted therapies [11–13]. Encouraging intracranial antitumor activity was observed with tucatinib treatment, a partial BBB penetration agent, in combination with trastuzumab and capecitabine in the HER2CLIMB study. However, the efficacy is balanced by major toxicities such as diarrhea and elevated liver enzymes [8, 14]. In the DESTINY-Breast03 study, T-DXd showed promising efficacy in patients with HER2+ MBC, including patients with stable BM. However, patients with LM or active BM were excluded from the study [5]. Recently, in the TUXEDO-1 trial, clinically relevant intracranial activity of T-DXd was observed in patients with untreated BM or BM progressing after local therapy, though the sample size was relatively small [15]. Therefore, targeted therapy with full BBB penetration and a good safety profile could potentially bring clinical benefits to patients. To this end, we designed DZD1516, an oral, potent, reversible, and selective HER2 TKI with full BBB penetration.

## Methods

Preclinical experiment methods, including establishment of BT474C1-Luci Mono1 cell clone, in vitro enzymatic assay, kinase panel assay, cell proliferation assay, establishment of SC model, BM and LM models, assessment of passive permeability and efflux ratio of P-gp and breast cancer resistance protein (BCRP) transporters, fraction unbound of DZD1516/DZ2678 in plasma and brain tissue, in vivo CNS-PK studies to evaluate CNS penetration of DZD1516/DZ2678, and immunohistochemical staining for pHER2 in xenograft tumor tissues are presented in Additional file 1. The corresponding statistical analysis methods are also presented in Additional file 1.

WEN-JI1 study (ClinicalTrials.gov: NCT04509596; Chinadrugtrial: CTR20202424) is a multi-center, open-label, phase 1 dose escalation study to assess the safety, tolerability, pharmacokinetics, and preliminary antitumor activity of DZD1516 in patients with HER2+ MBC who relapsed from standard of care in the USA and China. The study was conducted in accordance with the principles of the Declaration of Helsinki and Good Clinical Practice guidelines and with the approval of local institutional review boards/independent ethics committees at participating sites. All patients provided written informed consent to participate.

### Patient population

The study enrolled adult patients (age ≥ 18) with confirmed histological or cytological diagnosis of MBC and was HER2 positive. There were no limitations on the number of prior lines of systemic therapy; however, patients were required to have documented disease progression on the most recent disease evaluation. Additional eligibility criteria are provided in Additional file 1.

### Clinical study design

The maximum tolerated dose (MTD) of DZD1516 was defined based on safety data and the Bayesian optimal interval (BOIN) design (Additional file 1). The definition of DLT is provided in Additional file 1.

### Safety analysis

Safety evaluations were conducted throughout the study (Additional file 1).

The AE grading was assessed according to the NCI-Common Terminology Criteria for Adverse Events (CTCAE) (version 5.0) and was monitored until 28 days after the last dose.

### Efficacy analysis

Tumor imaging assessments were conducted every 6 weeks during the first 24 weeks (relative to C1D1) and every 9 weeks thereafter per investigator review according to modified RECIST v1.1 until progressive disease, starting a new anticancer therapy or withdrawal of informed consent (Additional file 1).

### Bioanalysis of plasma samples and pharmacokinetic assessments

Blood samples for PK analyses of DZD1516 and DZ2678 were collected on C0D1. Samples were obtained at pre-dose and at 0.5, 1, 1.5, 2, 2.5, 3, 4, 6, 8, 10, 24, 48, and 72 h after the dose, while for C1D15, samples were obtained at pre-dose and 0.5, 1, 1.5, 2, 2.5, 3, 4, 6, 8, and 10 h after the dose. CSF samples for PK analyses of DZD1516 and its metabolite DZ2678 were collected from patients with BM (C1D15) and LM (C1D15 and C3D1) at steady state (Additional file 1). Plasma and CSF samples were analyzed by LabCorp Madison laboratory (Madison, WI, USA) and LabCorp Shanghai laboratory (Shanghai, China) using a validated LC/MS/MS bioanalytical method to determine DZD1516 and DZ2678 concentrations. Non-compartmental PK analysis was performed using Phoenix WinNonlin (Certara USA Inc., version 8.3.3.33).

## Results

### Design and structure of DZD1516, which can selectively bind to the HER2 protein with optimal BBB penetration getting ***K***_p,uu_ to unity

**Fig. 1:**
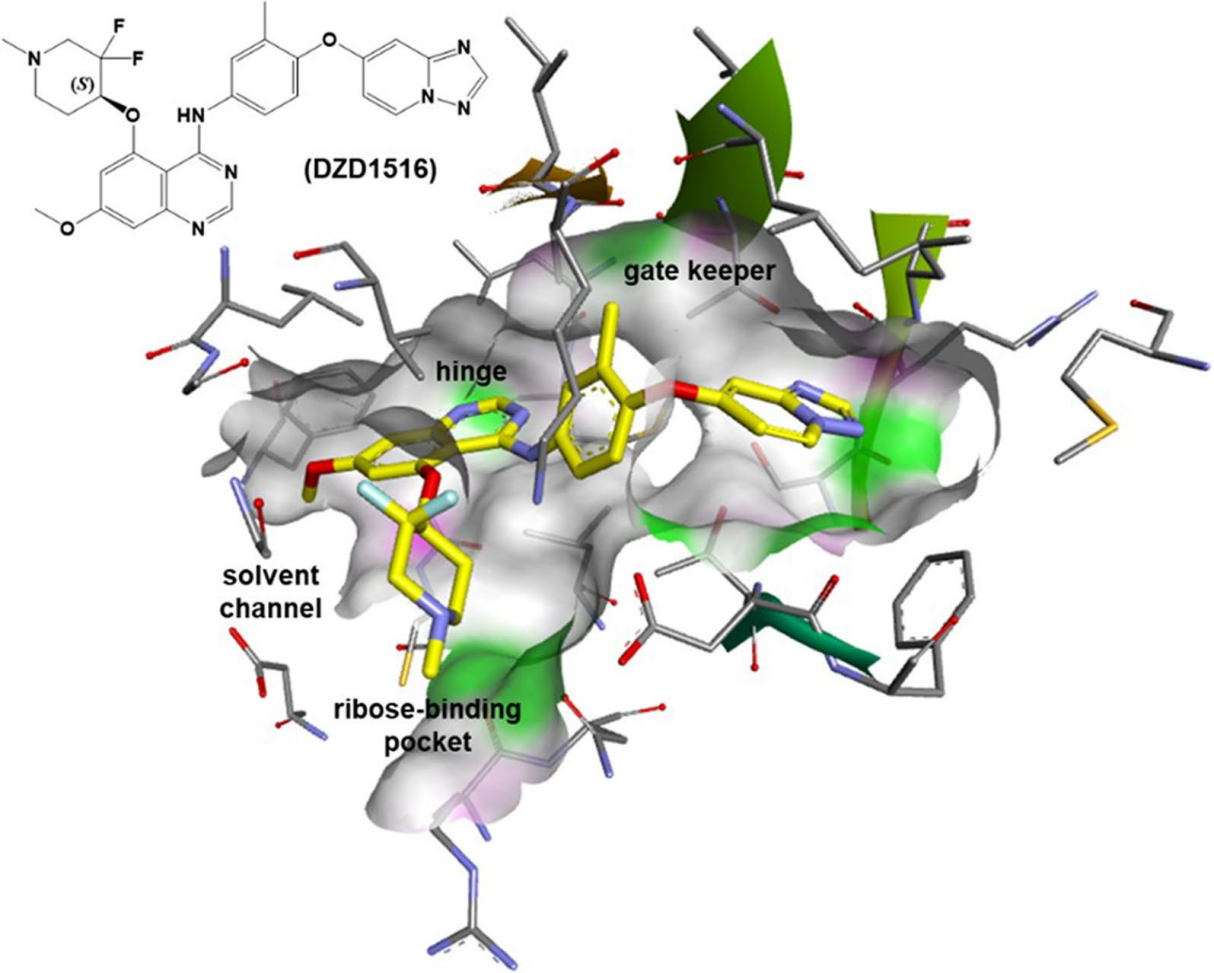
Modeling of DZD1516 with HER2 (PDB code: 3RCD). Key interactions of DZD1516 with HER2 protein include (i) H-bonding of quinazoline with hinge (Met801); (ii) piperidine occupied the ribose-binding pocket; (iii) [1, 2, 4]triazolo[1,5-a]pyridin-7-yloxy scaffold near the gatekeeper back pocket for HER2 selectivity. Yellow: carbon; purple: nitrogen; red: oxygen; light blue: fluorine. Colors on the protein surface represent the ATP binding pocket and are for clarity only

A discussion of the structure–activity relationship (SAR) cumulated in the design of DZD1516 (Fig. 1) is as follows. The HER2 inhibitory potency screening for SAR development was performed using HER2 and EGFR enzymatic assays. The cellular activity was evaluated in HER2+ cell line BT474C1 by measuring pHER2 and antiproliferation activities. Cellular transport assays measuring the permeability and efflux ratio of compounds in cells expressing human P-gp (MDR1) and BCRP were used to provide essential information for assessing their BBB penetration potential [16].

Strategically, a fragment-based approach was initiated to explore the quinazoline C5 position with substituents having a basic nitrogen atom projecting into the ribose-binding pocket of the ATP binding site. To this end, a series of oxygen-linked cyclic amines were synthesized (Additional file 2: Table S1). Among many different nitrogen-containing fragments that we have investigated, structures with a piperidine moiety were found promising. For example, the (*N*-methylpiperidin-4-yl)oxy moiety in Cpd-1 provided potent HER2 inhibition with cellular pHER2 IC_50_ of 18 nM. To our delight, Cpd-1 is also a non-P-gp transporter substrate with an efflux ratio of 1.5. It was believed that fluorine substitution could decrease the basicity of the piperidine nitrogen and impact the permeability and efflux of compounds across cell membranes. By combining the quinazoline scaffold with a 3,3-difluoro-*N*-methylpiperidine with an oxygen-linker as in Cpd-2 (pHER2 IC_50_ = 5.8 nM), a threefold improvement of cellular potency was achieved. Cpd-2 was found to maintain the HER2 target potency and efflux ratio in the P-gp and BCRP transporter assays less than two thresholds. Therefore, Cpd-2 has the potential to cross the human BBB efficiently for not being a substrate of both the human P-gp and BCRP transporters. Unfortunately, Cpd-2 was found not suitable to progress for development as it was quickly metabolized by human hepatocytes with a clearance of Clint = 128 [(µL/min)/(10^6^ cells)].

Further optimization effort focused on the 5-((3,3-difluoro-1-methylpiperidin-4-yl)oxy) quinazoline scaffold to achieve a balance between potency, selectivity, brain penetration, and pharmacokinetic properties. During this optimization stage, substituents at the C6/C7 positions were explored for analogs with better metabolite stability while maintaining the previously optimized HER2 potency and brain penetration properties. In short, a simple C-7 methoxy group as in Cpd-5 was identified as having an improved human hepatocytes clearance with Clint = 12 [(µL/min)/(10^6^ cells)], presumably by blocking the oxidation on the quinazoline core. Based on the preferred HER2 target potency, the (S)-enantiomer Cpd-5 was selected for in vitro, in vivo efficacy, and safety studies.

Overall, Cpd-5 (designated as DZD1516) was a highly cellular potent HER2 inhibitor. Since low CNS penetration is associated with efflux, avoiding active efflux pumps (e.g., P-gp and BCRP) of therapeutics at BBB will improve brain exposure. DZD1516 was optimized as a non-P-gp and non-BCRP substrate (efflux ratio < 2) with high intrinsic passive permeability (Caco-2 Papp = 90 × 10^−6^ cm/s) to facilitate brain penetration. Since a *K*_p,uu_ < 1 will reduce unbound brain levels relative to unbound plasma levels, raising a low *K*_p,uu_ value toward unity will be beneficial for brain exposure. In vivo, *K*_p,uu,brain_ and *K*_p,uu,CSF_ in rat ranged from 0.57 to 0.87 for DZD1516 and 0.12 to 0.23 for DZ2678. These data indicated approximately equivalent exposure of unbound DZD1516 among brain tissue, CSF, and plasma, while DZD2678 exhibited less CNS penetration than DZD1516. Similar data were observed in CNS penetration assessment in monkey, where DZD1516 (*K*_p,uu,CSF_ = 4.1) demonstrated better CNS entry than DZ2678 (*K*_p,uu,CSF_ = 0.865). With proper human intrinsic hepatocytes clearance, DMPK modeling predicted a human half-life of about 8 h (hrs), suitable for twice daily dosing. DZD1516 also behaved favorably in numerous in vitro safety pharmacology studies, such as kinases panel selectivity, not a CYP P450s inhibitor and inducer, and potassium ion-channel hERG IC_50_ = 3.29 µM.

Finally, the N-demethylated analog of DZD1516 was also synthesized. Cpd-6 (designated as DZ2678) was observed as the major metabolite in in vivo rat, mouse, dog, and monkey pharmacokinetic studies. Overall, DZ2678 shared very similar biological and DMPK properties compared with DZD1516.

### DZD1516 is a potent and selective HER2 inhibitor

DZD1516 and its active metabolite DZ2768 showed potent and comparable inhibition with IC_50_ of 0.56 nM and 0.95 nM, respectively, against HER2 in the enzymatic assay (Additional file 3: Fig. S1A).

The kinome selectivity profile of DZD1516 was tested in in vitro kinase assay of 121 recombinant human kinases (Additional file 3: Fig. S1B). The results showed that only 12 kinases were inhibited > 50%, and IC_50_ of these kinases inhibited further detected (Additional file 2: Table S2). Overall, DZD1516 only displayed potent inhibitory activity against HER2 kinase, with limited off-target activity against the rest of the kinome.

DZD1516 showed potent activity in downregulating pHER2 with IC_50_ of 4.4 nM in BT474C1 cell line overexpressing HER2 (Additional file 3: Fig. S1C). The effect of DZD1516 on wild-type EGFR was assessed by measuring pEGFR in A431 cell line that overexpressed wild-type EGFR, which was used to test various clinically approved EGFR TKIs [17, 18]. DZD1516 showed poor activity in downregulating pEGFR with IC_50_ of 1455 nM in A431 cells, showing greater than 300-fold selectivity against wild-type EGFR (Additional file 3: Fig. S1C). DZD1516 potently suppresses cell proliferation with GI_50_ of 20 nM in cells overexpressing HER2 but has feeble activity in A431 cells with GI_50_ of 8867 nM (Additional file 3: Fig. S1D). DZ2678 showed similar potency and selectivity profiles to DZD1516 (Additional file 2: Table S1).

### DZD1516 monotherapy or in combination with HER2 ADC demonstrated profound antitumor activities in BM, LM, and subcutaneous models in nude mice

An agent that can control both intracranial and extracranial tumors is desirable to be an efficacious drug for treating breast cancer. We established subcutaneous (SC), BM, and LM models in nude mice (Additional file 1, Additional file 4: Fig. S2) to test the hypothesis that DZD1516 could effectively control both intracranial and extracranial tumors.

**Fig. 2:**
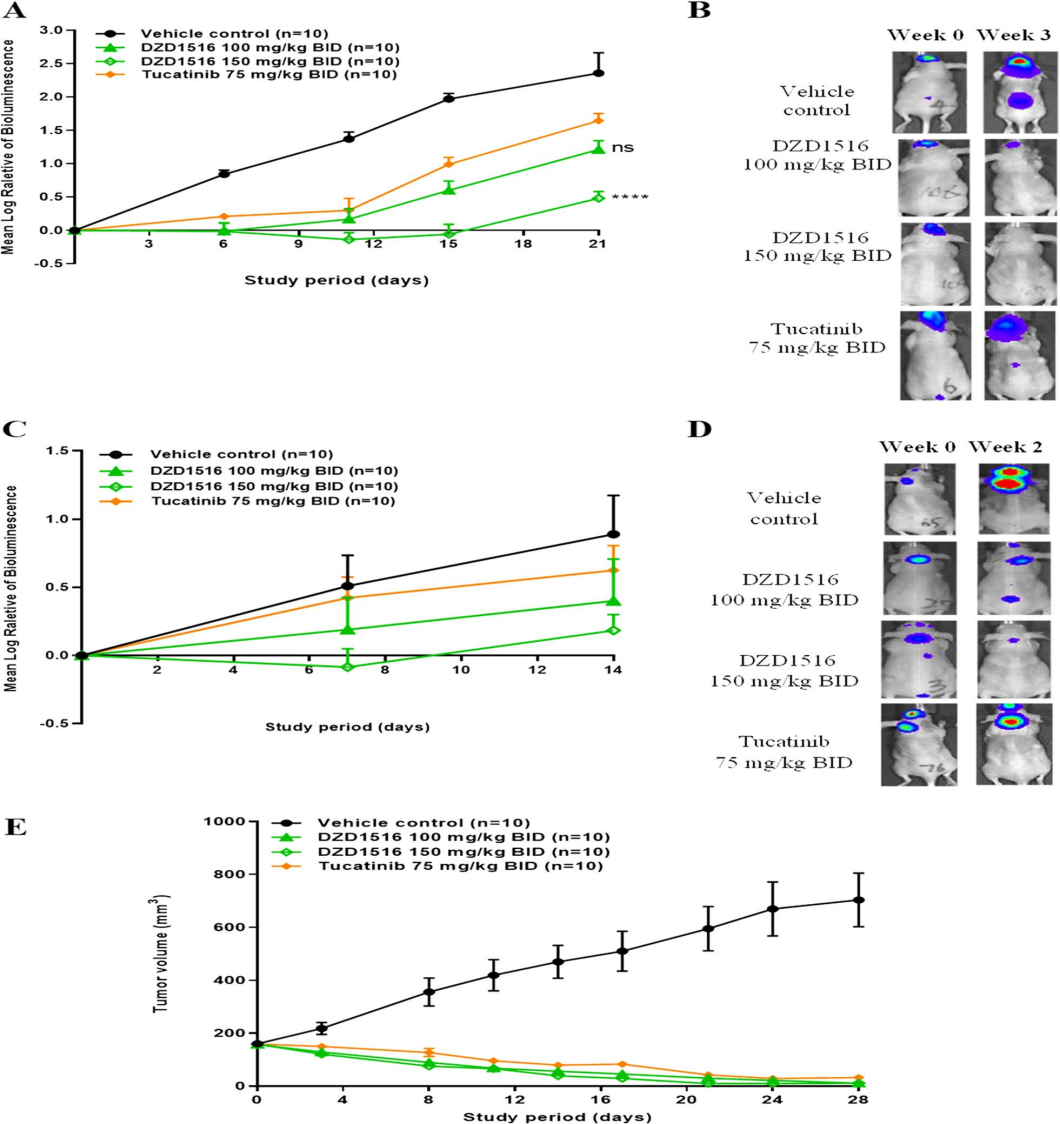
Antitumor activity of DZD1516 in BM, LM, and subcutaneous BT474C1-Luci Mono1 xenograft mice model. The BT474C1-Luci Mono1 cell model was a stable clone generated by transfection of luciferase gene into BT474 cell line and selection of stable clones. The mice were treated with DZD1516 at 100 mg/kg and 150 mg/kg twice daily, respectively, after tumors were established. **A** Plots of tumor volume in BM model. The statistical analysis of tumor volume difference at week 3 between DZD1516 and tucatinib groups was performed by two-way ANOVA, compared with the tucatinib group. ****P < 0.0001, ns: not significant. **B** Representative images of bioluminescent signals at week 0 and week 3 after the start of compound treatment in BM model. **C** Plots of tumor volume in LM model. **D** Representative images of bioluminescent signals at week 0 and week 2 after the start of compound treatment in LM model. **E** Plots of tumor volume in subcutaneous model. *BM* brain metastasis, *LM* leptomeningeal metastasis. BID: twice daily

In the BM model which was established by intracerebral implantation of tumor cells, DZD1516 produced a significant antitumor effect with 48% TGI and 79% TGI at 100 mg/kg and 150 mg/kg, respectively (Fig. 2A). The antitumor activity of DZD1516 at 150 mg/kg was significantly better than that of tucatinib at 75 mg/kg, its MTD in mice (*P* < 0.0001). To visually reflect such a profound antitumor efficacy, the representative images of bioluminescent signals at week 0 and week 3 after treatment are shown in Fig. 2B.

In the LM model, DZD1516 generated a potent antitumor growth effect with 57% TGI and 81% TGI at 100 mg/kg and 150 mg/kg, respectively (Fig. 2C). The efficacy of DZD1516 was numerically better than that of tucatinib. The representative images of bioluminescent signals at week 2 after treatment are shown in Fig. 2D.

In the SC model, DZD1516 induced a dose-dependent antitumor efficacy (Fig. 2E). The tumor remission was achieved at 100 mg/kg or 150 mg/kg, which was equally effective as tucatinib.

Given the current clinical practice that the combination therapy was the standard treatment for HER2+ breast cancer, we evaluated the potential of the combination of DZD1516 with different anti-HER2 agents, especially HER2 ADCs, in animal models.

**Fig. 3:**
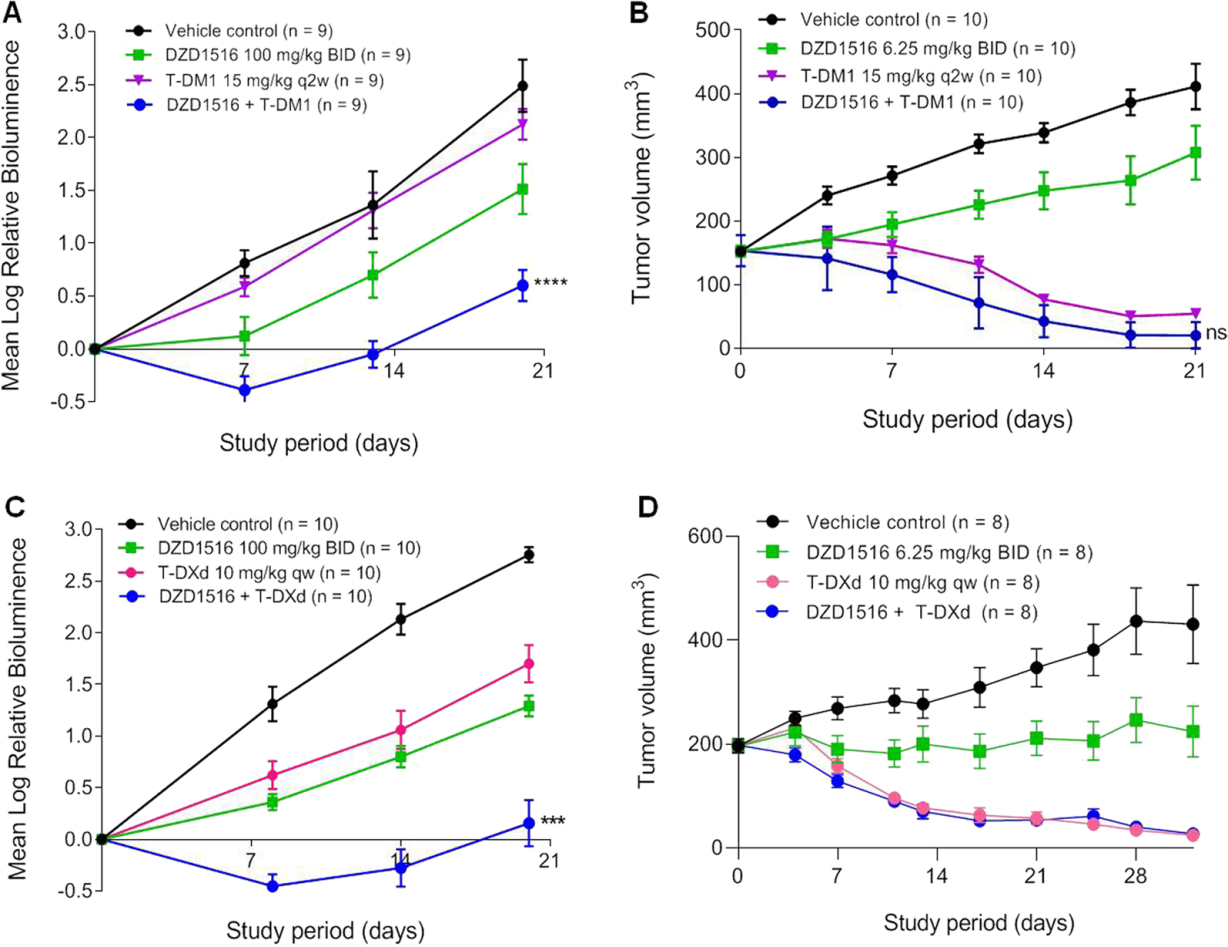
Antitumor activity of DZD1516 in combination with T-DM1 or T-DXd in BT474C1-Luci Mono1 xenograft mice model. The BT474C1-Luci Mono1 cell model was a stable clone generated by transfection of luciferase gene into BT474 cell line and selection of stable clones. **A** Efficacy study of DZD1516 in combination with T-DM1 in the BM model. *n* = 9/group. ****P < 0.0001 (vs. T-DM1 group, two-way ANOVA). **B** Efficacy study of DZD1516 in combination with T-DM1 in the SC model. *n* = 10/group. ns: not significant (vs. T-DM1 group, two-way ANOVA). **C** Efficacy study of DZD1516 in combination with T-DXd in the BM model. ***P < 0.001 (vs. T-DXd group, two-way ANOVA). **D** Efficacy study of DZD1516 in combination with T-DXd in the SC xenograft model. BID: twice daily; q2w: once every 2 weeks. qw: once weekly. *BM* brain metastasis, *SC* subcutaneous

The antitumor effect of DZD1516 in combination with T-DM1 was assessed in BM and SC models. In the BM model, DZD1516 was dosed at 100 mg/kg twice daily, and T-DM1 was dosed at its MTD (15 mg/kg once every 2 weeks). In the SC model, a lower dose (6.25 mg/kg) of DZD1516 was chosen for the combination study to ensure enough window to evaluate the potential synergistic effect, given that DZD1516 monotherapy at 50 mg/kg already induced tumor shrinkage or complete tumor remission in the subcutaneous xenograft model (data not shown). DZD1516 in combination with T-DM1 showed numerically better antitumor activity than T-DM1 alone as shown in Fig. 3A, B.

The antitumor effect of DZD1516 in combination with another HER2 ADC, T-DXd, was also assessed in BM and SC models. T-Dxd was dosed at MTD (10 mg/kg once weekly). In the BM model, as shown in Fig. 3C, DZD1516 combined with T-DXd produced significantly better efficacy than T-DXd or DZD1516 alone. As shown in Fig. 3D, the combination induced complete tumor remission in the SC model. However, as the T-DXd alone already resulted in complete tumor remission, it requires conducting further study with a lower dose of T-DXd to address whether there was a synergistic antitumor effect or not with the combination in the subcutaneous setting.

The combination groups showed similar body weight change compared to either single-agent DZD1516 or single-agent ADCs (Additional file 5: Fig. S3). In addition, there were no reported in-life gross signs of toxicity, such as diarrhea and skin rash.

### DZD1516 showed good pharmacokinetic (PK) and pharmacodynamic (PD) correlation in HER2-positive SC model

**Fig. 4:**
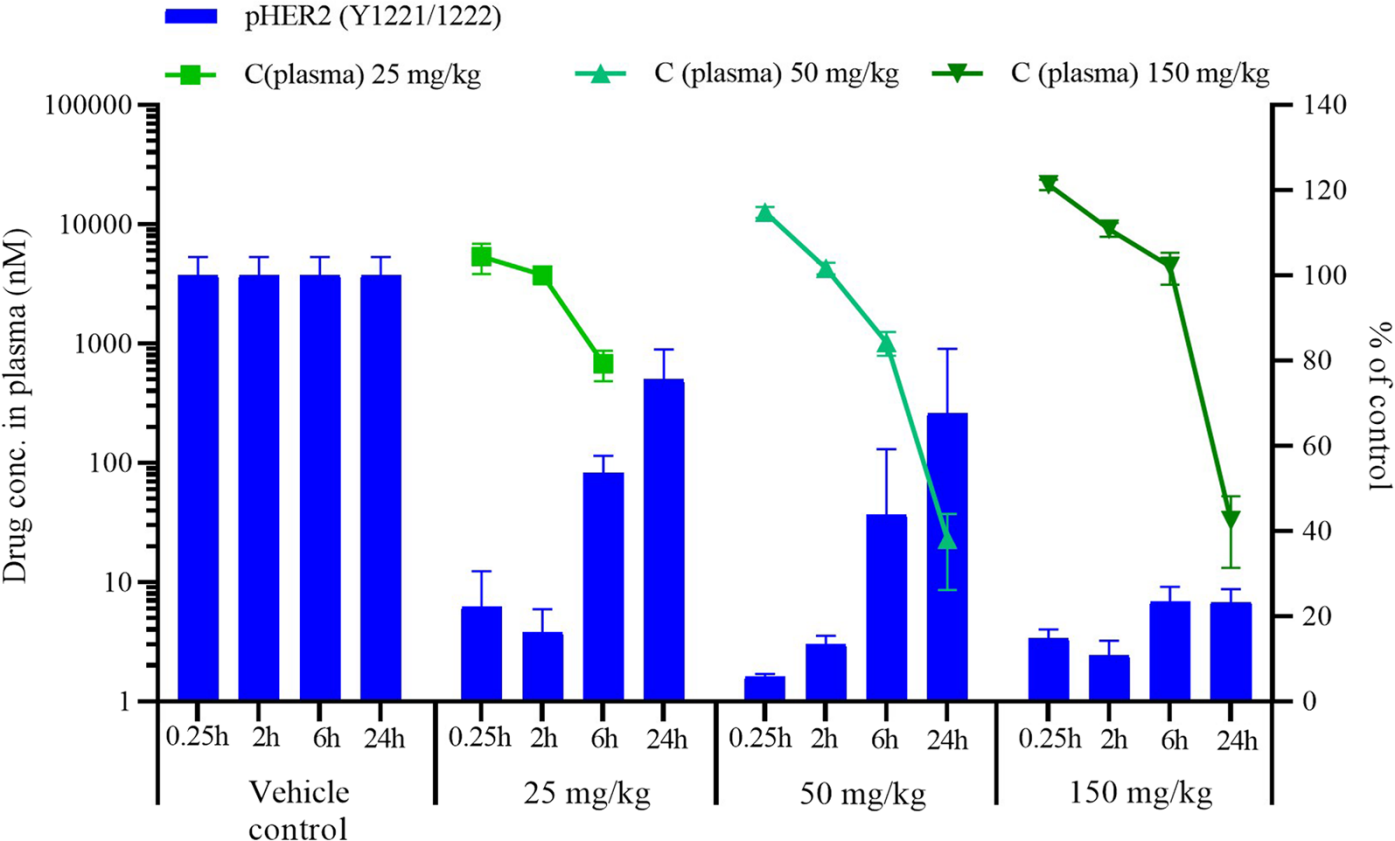
PK and PD relationship post-single dose of DZD1516 in BT474C1-Luci Mono1 SC xenograft mice model. A single dosing of DZD1516 at 25, 50, 150 mg/kg was administrated when the tumor volume reached 200–600 mm^3^. Plasma and tumor tissues were collected at 0.25, 2, 6, and 24 h post-dosing to analyze the PK/PD correlation. The pHER2 expression in tumor tissues was detected by immunohistochemistry (IHC) and normalized to the vehicle control group. Each time point had tumor tissues from three mice to detect the pHER2 signal. *PK* pharmacokinetics, *PD* pharmacodynamics, *SC* subcutaneous

Dose-dependent pHER2 inhibition was observed with DZD1516 treatment. At 50 mg/kg, DZD1516 led to more than 94% inhibition of pHER2 as early as at 0.25 h, and the inhibition effect was maintained for 6 h. Besides, a higher dose at 150 mg/kg led to 89% of pHER2 inhibition, and the effect lasted for 24 h (Fig. 4). The IHC representative images of pHER2 post-single dosing of DZD1516 in SC xenograft tissues are shown in Additional file 6: Fig. S4. Higher doses of DZD1516 led to higher exposure in plasma and concurrent more profound inhibition of pHER2 in tumor xenograft tissue, suggesting a positive correlation between drug exposure and pHER2 inhibition in the HER2+ xenograft model (Fig. 4).

### Phase 1 clinical study of DZD1516 in patients with HER2+ metastatic breast cancer

Between September 21, 2020, and August 29, 2022 (DCO), twenty-four patients with HER2 + metastatic breast cancer were enrolled; among them, twenty-three were dosed with DZD1516 monotherapy. DZD1516 dose levels ranged from 25 to 300 mg twice daily (BID).

Patient demographics and baseline characteristics are summarized in Table 1. Fifteen patients (65.2%) had CNS metastases at the study entry. Most patients were heavily pre-treated, with median seven lines of prior systemic therapies. All patients had been treated with HER2 antibodies or ADCs. Nineteen patients (82.6%) had also received prior HER2 TKI treatment.

**Table 1:** Summary of patient demographics and baseline characteristics

	25 mg (*N* = 1)	50 mg (*N* = 4)	100 mg (*N* = 4)	200 mg (*N* = 5)	250 mg (*N* = 5)	300 mg (*N* = 4)	Total (N = 23)
Age (year)							
Median	64	57.5	50.0	61.0	57.0	42.0	57.0
Min, Max	64, 64	47, 63	36, 61	46, 66	38, 71	39, 63	36, 71
Age group, *n* (%)							
< 60	0 (0.0)	3 (75.0)	2 (50.0)	2 (40.0)	3 (60.0)	3 (75.0)	13 (56.5)
≥ 60	1 (100.0)	1 (25.0)	2 (50.0)	3 (60.0)	2 (40.0)	1 (25.0)	10 (43.5)
Race, *n* (%)							
Asian	1 (100.0)	3 (75.0)	2 (50.0)	5 (100.0)	3 (60.0)	3 (75.0)	17 (73.9)
White	0 (0.0)	0 (0.0)	2 (50.0)	0 (0.0)	2 (40.0)	0 (0.0)	4 (17.4)
Other	0 (0.0)	1 (25.0)	0 (0.0)	0 (0.0)	0 (0.0)	1 (25.0)	2 (8.7)
Metastatic sites, *n* (%)	1 (100.0)	4 (100.0)	4 (100.0)	5 (100.0)	5 (100.0)	4 (100.0)	23 (100.0)
With BM, *n* (%)	0 (0.0)	4 (100.0)	3 (75.0)	2 (40.0)	3 (60.0)	2 (50.0)	14 (60.9)
With LM, *n* (%)	0 (0.0)	0 (0.0)	1 (25.0)	0 (0.0)	0 (0.0)	0 (0.0)	1 (4.3)
Without CNS, *n* (%)	1 (100.0)	0 (0.0)	0 (0.0)	3 (60.0)	2 (40.0)	2 (50.0)	8 (34.8)
ECOG, *n* (%)							
0	0 (0.0)	1 (25.0)	0 (0.0)	0 (0.0)	1 (20.0)	1 (25.0)	3 (13.0)
1	1 (100.0)	3 (75.0)	4 (100.0)	5 (100.0)	4 (80.0)	3 (75.0)	20 (87.0)
With surgical therapy, *n* (%)	1 (100.0)	4 (100.0)	3 (75.0)	5 (100.0)	5 (100.0)	3 (75.0)	21 (91.3)
With radiation therapy (intracranial and/or extracranial), *n* (%)	1 (100.0)	4 (100.0)	4 (100.0)	5 (100.0)	3 (60.0)	3 (75.0)	20 (87.0)
With any prior systemic anticancer therapy, *n* (%)	1 (100.0)	4 (100.0)	4 (100.0)	5 (100.0)	5 (100.0)	4 (100.0)	23 (100.0)
Median	7.0	9.0	5.5	9.0	8.0	5.5	7.0
Min, max	7, 7	3, 15	4, 8	4, 10	6, 11	3, 9	3, 15
Therapy class, *n* (%)							
HER2 antibody and/or ADC	1 (100.0)	4 (100.0)	4 (100.0)	5 (100.0)	5 (100.0)	4 (100.0)	23 (100.0)
HER2 TKI	0 (0.0)	3 (75.0)	3 (75.0)	5 (100.0)	4 (80.0)	4 (100.0)	19 (82.6)
Chemotherapy	1 (100.0)	4 (100.0)	4 (100.0)	5 (100.0)	5 (100.0)	4 (100.0)	23 (100.0)
Endocrine therapy	1 (100.0)	2 (50.0)	2 (50.0)	3 (60.0)	4 (80.0)	2 (50.0)	14 (60.9)
Other	0 (0.0)	2 (50.0)	1 (25.0)	3 (60.0)	5 (100.0)	2 (50.0)	13 (56.5)

*N* number of subjects in the analysis set for each treatment group, *Max* maximum, *Min* minimum, *BM* brain metastasis, *LM* leptomeningeal metastasis, *ADC* antibody–drug conjugate, *TKI* tyrosine kinase inhibitor. The percentage was calculated based on N as the denominator. Patients with BM are defined as BM regardless of with or without extracranial metastases. Patients with LM are defined as LM regardless of the patients are with or without extracranial/brain metastases

Dose escalation proceeded through the planned 25–200 mg cohorts, with no dose-limiting toxicity (DLT) observed. In the 300 mg cohort, two out of four patients experienced DLTs (one was grade 3 musculoskeletal pain, and another one was grade 2 adverse events including headache, nausea, vomiting, fatigue, etc., leading to dose interruption for 20 days). As a result, the 300 mg dose was assessed as intolerable, and the 250 mg BID cohort was open for enrollment as agreed by the safety review committee (SRC). With none of the patients in the 250 mg cohort experiencing DLTs, the 250 mg BID dose was defined as the MTD of DZD1516.

Across all dose levels, the median duration of treatment was 1.5 months, with the longest duration of treatment close to 3 months. Grade 3 adverse events (AEs) were reported in 6 patients (26.1%) (Table 2). According to investigators’ judgment, treatment-related grade 3 AEs in 4 patients (17.4%) were considered drug-related. AEs leading to dose interruption and reduction were reported in 5 (21.7%) and 3 (13.0%) patients, respectively, and majority were in the 300 mg cohort. DZD1516 was resumed with tolerability at the initial or lower doses. No grade 4 or 5 AEs were reported.

**Table 2:** Overall summary of TEAE

*n* (%)	25 mg (*N* = 1)	50 mg (*N* = 4)	100 mg (*N* = 4)	200 mg (*N* = 5)	250 mg (*N* = 5)	300 mg (*N* = 4)	Total (*N* = 23)
Subjects with any TEAE	1 (100.0)	3 (75.0)	4 (100.0)	5 (100.0)	5 (100.0)	4 (100.0)	22 (95.7)
Grade 3^a^	0 (0.0)	0 (0.0)	1 (25.0)	1 (20.0)	1 (20.0)	3 (75.0)	6 (26.1)
SAE	0 (0.0)	0 (0.0)	1 (25.0)	0 (0.0)	1 (20.0)	0 (0.0)	2 (8.7)
Leading to treatment interruption	0 (0.0)	0 (0.0)	1 (25.0)	0 (0.0)	1 (20.0)	3 (75.0)	5 (21.7)
Leading to dose reduction	0 (0.0)	0 (0.0)	0 (0.0)	0 (0.0)	0 (0.0)	3 (75.0)	3 (13.0)
Leading to treatment discontinuation	0 (0.0)	0 (0.0)	0 (0.0)	0 (0.0)	0 (0.0)	1 (25.0)	1 (4.3)
Leading to death	0 (0.0)	0 (0.0)	0 (0.0)	0 (0.0)	0 (0.0)	0 (0.0)	0 (0.0)
Subjects with any drug-related TEAE^b^	1 (100.0)	3 (75.0)	4 (100.0)	5 (100.0)	4 (80.0)	4 (100.0)	21 (91.3)
Grade 3^a^	0 (0.0)	0 (0.0)	1 (25.0)	1 (20.0)	0 (0.0)	2 (50.0)	4 (17.4)
SAE	0 (0.0)	0 (0.0)	1 (25.0)	0 (0.0)	0 (0.0)	0 (0.0)	1 (4.3)
Leading to treatment interruption	0 (0.0)	0 (0.0)	1 (25.0)	0 (0.0)	0 (0.0)	2 (50.0)	3 (13.0)
Leading to dose reduction	0 (0.0)	0 (0.0)	0 (0.0)	0 (0.0)	0 (0.0)	3 (75.0)	3 (13.0)
Leading to treatment discontinuation	0 (0.0)	0 (0.0)	0 (0.0)	0 (0.0)	0 (0.0)	0 (0.0)	0 (0.0)
Leading to death	0 (0.0)	0 (0.0)	0 (0.0)	0 (0.0)	0 (0.0)	0 (0.0)	0 (0.0)

*AE* adverse event, *CTCAE* Common Terminology Criteria for Adverse Events, *N* number of subjects in the analysis set for each treatment group, *SAE* Serious Adverse Event, *TEAE* treatment-emergent adverse event. The percentage was calculated based on N as the denominator

^a^Adverse event grades were evaluated by investigators according to CTCAE version 5.0

^b^AEs with missing relationships were reported as related

Common AEs are summarized in Table 3. The majority of AEs were CTCAE grade 1 and were reversible. The most common AEs (of any grades) were headache, vomiting, and hemoglobin decreased. No diarrhea or skin rash of any grade was observed at the MTD or lower doses. A single case of grade 2 diarrhea was reported in the 300 mg cohort and resolved two days later without supportive care.

**Table 3:** Summary of Common (≥ 10%) TEAEs by MedDRA System Organ Class, Preferred Term and CTCAE Grade

System organ class preferred term	25 mg (*N* = 1) *n* (%)		50 mg (*N* = 4) *n* (%)		100 mg (*N* = 4) *n* (%)		200 mg (*N* = 5) *n* (%)		250 mg (*N* = 5) *n* (%)		300 mg (*N* = 4) *n* (%)		Total (*N* = 23) n (%)	
	Any Grade	≥ Grade 3	Any Grade	≥ Grade 3	Any Grade	≥ Grade 3	Any Grade	≥ Grade 3	Any Grade	≥ Grade 3	Any Grade	≥ Grade 3	Any Grade	≥ Grade 3
Participants with any TEAE	1 (100.0)	0 (0.0)	3 (75.0)	0 (0.0)	4 (100.0)	1 (25.0)	5 (100.0)	1 (20.0)	5 (100.0)	1 (20.0)	4 (100.0)	3 (75.0)	22 (95.7)	6 (26.1)
Investigations	1 (100.0)	0 (0.0)	3 (75.0)	0 (0.0)	3 (75.0)	1 (25.0)	4 (80.0)	1 (20.0)	2 (40.0)	0 (0.0)	1 (25.0)	0 (0.0)	14 (60.9)	2 (8.7)
Hemoglobin decreased	0 (0.0)	0 (0.0)	2 (50.0)	0 (0.0)	0 (0.0)	0 (0.0)	3 (60.0)	0 (0.0)	2 (40.0)	0 (0.0)	0 (0.0)	0 (0.0)	7 (30.4)	0 (0.0)
Aspartate aminotransferase increased	0 (0.0)	0 (0.0)	1 (25.0)	0 (0.0)	0 (0.0)	0 (0.0)	1 (20.0)	0 (0.0)	2 (40.0)	0 (0.0)	1 (25.0)	0 (0.0)	5 (21.7)	0 (0.0)
Blood bilirubin increased	1 (100.0)	0 (0.0)	0 (0.0)	0 (0.0)	0 (0.0)	0 (0.0)	2 (40.0)	0 (0.0)	1 (20.0)	0 (0.0)	0 (0.0)	0 (0.0)	4 (17.4)	0 (0.0)
Alanine aminotransferase increased	0 (0.0)	0 (0.0)	1 (25.0)	0 (0.0)	0 (0.0)	0 (0.0)	0 (0.0)	0 (0.0)	1 (20.0)	0 (0.0)	1 (25.0)	0 (0.0)	3 (13.0)	0 (0.0)
Lipase increased	0 (0.0)	0 (0.0)	2 (50.0)	0 (0.0)	0 (0.0)	0 (0.0)	1 (20.0)	0 (0.0)	0 (0.0)	0 (0.0)	0 (0.0)	0 (0.0)	3 (13.0)	0 (0.0)
Platelet count decreased	0 (0.0)	0 (0.0)	1 (25.0)	0 (0.0)	1 (25.0)	0 (0.0)	1 (20.0)	0 (0.0)	0 (0.0)	0 (0.0)	0 (0.0)	0 (0.0)	3 (13.0)	0 (0.0)
White blood cell count decreased	0 (0.0)	0 (0.0)	1 (25.0)	0 (0.0)	0 (0.0)	0 (0.0)	0 (0.0)	0 (0.0)	2 (40.0)	0 (0.0)	0 (0.0)	0 (0.0)	3 (13.0)	0 (0.0)
Nervous system disorders	0 (0.0)	0 (0.0)	1 (25.0)	0 (0.0)	3 (75.0)	0 (0.0)	2 (40.0)	0 (0.0)	4 (80.0)	0 (0.0)	4 (100.0)	2 (50.0)	14 (60.9)	2 (8.7)
Headache	0 (0.0)	0 (0.0)	1 (25.0)	0 (0.0)	2 (50.0)	0 (0.0)	1 (20.0)	0 (0.0)	2 (40.0)	0 (0.0)	4 (100.0)	0 (0.0)	10 (43.5)	0 (0.0)
Dizziness	0 (0.0)	0 (0.0)	0 (0.0)	0 (0.0)	1 (25.0)	0 (0.0)	0 (0.0)	0 (0.0)	1 (20.0)	0 (0.0)	1 (25.0)	1 (25.0)	3 (13.0)	1 (4.3)
Metabolism and nutrition disorders	0 (0.0)	0 (0.0)	1 (25.0)	0 (0.0)	3 (75.0)	0 (0.0)	3 (60.0)	0 (0.0)	1 (20.0)	0 (0.0)	3 (75.0)	0 (0.0)	11 (47.8)	0 (0.0)
Decreased appetite	0 (0.0)	0 (0.0)	0 (0.0)	0 (0.0)	1 (25.0)	0 (0.0)	0 (0.0)	0 (0.0)	0 (0.0)	0 (0.0)	3 (75.0)	0 (0.0)	4 (17.4)	0 (0.0)
Gastrointestinal disorders	0 (0.0)	0 (0.0)	0 (0.0)	0 (0.0)	2 (50.0)	1 (25.0)	3 (60.0)	0 (0.0)	1 (20.0)	1 (20.0)	4 (100.0)	1 (25.0)	10 (43.5)	3 (13.0)
Vomiting	0 (0.0)	0 (0.0)	0 (0.0)	0 (0.0)	2 (50.0)	1 (25.0)	3 (60.0)	0 (0.0)	0 (0.0)	0 (0.0)	3 (75.0)	1 (25.0)	8 (34.8)	2 (8.7)
Nausea	0 (0.0)	0 (0.0)	0 (0.0)	0 (0.0)	0 (0.0)	0 (0.0)	1 (20.0)	0 (0.0)	1 (20.0)	0 (0.0)	4 (100.0)	1 (25.0)	6 (26.1)	1 (4.3)
Constipation	0 (0.0)	0 (0.0)	0 (0.0)	0 (0.0)	1 (25.0)	0 (0.0)	1 (20.0)	0 (0.0)	0 (0.0)	0 (0.0)	1 (25.0)	0 (0.0)	3 (13.0)	0 (0.0)
General disorders and administration site conditions	0 (0.0)	0 (0.0)	0 (0.0)	0 (0.0)	1 (25.0)	0 (0.0)	2 (40.0)	0 (0.0)	2 (40.0)	0 (0.0)	3 (75.0)	2 (50.0)	8 (34.8)	2 (8.7)
Fatigue	0 (0.0)	0 (0.0)	0 (0.0)	0 (0.0)	1 (25.0)	0 (0.0)	1 (20.0)	0 (0.0)	1 (20.0)	0 (0.0)	1 (25.0)	1 (25.0)	4 (17.4)	1 (4.3)
Malaise	0 (0.0)	0 (0.0)	0 (0.0)	0 (0.0)	0 (0.0)	0 (0.0)	1 (20.0)	0 (0.0)	1 (20.0)	0 (0.0)	1 (25.0)	1 (25.0)	3 (13.0)	1 (4.3)
Musculoskeletal and connective tissue disorders	0 (0.0)	0 (0.0)	1 (25.0)	0 (0.0)	0 (0.0)	0 (0.0)	0 (0.0)	0 (0.0)	3 (60.0)	1 (20.0)	3 (75.0)	1 (25.0)	7 (30.4)	2 (8.7)
Renal and urinary disorders	0 (0.0)	0 (0.0)	1 (25.0)	0 (0.0)	1 (25.0)	0 (0.0)	2 (40.0)	0 (0.0)	0 (0.0)	0 (0.0)	1 (25.0)	0 (0.0)	5 (21.7)	0 (0.0)
Proteinuria	0 (0.0)	0 (0.0)	1 (25.0)	0 (0.0)	0 (0.0)	0 (0.0)	2 (40.0)	0 (0.0)	0 (0.0)	0 (0.0)	1 (25.0)	0 (0.0)	4 (17.4)	0 (0.0)
Blood and lymphatic system disorders	0 (0.0)	0 (0.0)	0 (0.0)	0 (0.0)	1 (25.0)	0 (0.0)	1 (20.0)	0 (0.0)	1 (20.0)	0 (0.0)	0 (0.0)	0 (0.0)	3 (13.0)	0 (0.0)
Anemia	0 (0.0)	0 (0.0)	0 (0.0)	0 (0.0)	1 (25.0)	0 (0.0)	1 (20.0)	0 (0.0)	1 (20.0)	0 (0.0)	0 (0.0)	0 (0.0)	3 (13.0)	0 (0.0)
Cardiac disorders	0 (0.0)	0 (0.0)	0 (0.0)	0 (0.0)	0 (0.0)	0 (0.0)	1 (20.0)	0 (0.0)	2 (40.0)	0 (0.0)	0 (0.0)	0 (0.0)	3 (13.0)	0 (0.0)

*N* number of participants in the analysis set for each treatment group, *TEAE* treatment-emergent adverse event. MedDRA version 24.1 was used for coding. The percentage was calculated based on N as denominator. For number of participants: participants with multiple events within a given preferred term and system organ class were counted only once for each preferred term and system organ class, respectively

The incidence and grade of AEs seem to be dose-related. Treatment-related headaches were reported only in the ≥ 200 mg cohorts. However, due to the small number of patients at each dose level, no significant relationship between dose level and AE grades could be defined.

The pharmacokinetics of DZD1516 and metabolite DZ2678 have been characterized in patients with breast cancer. Following single oral administration from 50 to 300 mg, DZD1516 was absorbed with a median *t*_max_ of 2.15–3.22 h. Furthermore, steady-state drug exposure of DZD1516 appeared to be achieved by 15 days of twice daily dosing, resulting in an accumulation of approximately twofold, which is expected based on its half-life of 13.3–20.1 h. DZ2678 exhibited a similar PK profile as that of DZD1516 but with overall lower exposure.

Similar to DZD1516, circulating metabolite DZ2678 is pharmacologically active against HER2 target with similar potency as DZD1516 in vitro. DZ2678 exposure is 38%–71% of DZD1516 at steady state. The combined pharmacology effects of DZD1516 and DZ2678 were utilized when assessing the antitumor activity of the compound. The combined molar exposure (area under the plasma concentration–time curve) of DZD1516 and DZ2678 increased approximately in a dose-proportional manner from 50 to 250 mg after multiple twice daily dosing. Steady-state trough concentrations of DZD1516 and DZ2678 at ≥ 100 mg dose levels are above in vitro IC_50_ of BT474C1 cells, suggesting sustained inhibition of pHER2 signaling pathways.

Considering DZD1516 and DZ2678 are not substrate of P-gp and/or BCRP, *K*_p,uu,CSF_ can be used as a surrogate biomarker for CNS penetration assessment. In patients with CNS metastases, *K*_p,uu,CSF_ of DZD1516 and DZ2678 was around 2.1 and 0.76, respectively, suggesting effective CNS penetration of both parent and metabolite in human.

Twenty-one patients had completed at least one post-treatment tumor assessment. With median seven lines of prior systemic treatment, per investigators’ assessment, the best antitumor efficacy in intracranial, extracranial, and overall lesions were stable disease (Additional file 7: Fig. S5). A total of 6 out of 23 patients achieved stable disease. At the MTD (250 mg), the disease control rate was 60% (3/5). All patients have discontinued from treatment at the DCO, and the main reason for discontinuation was disease progression. Among patients with CNS metastases at study entry and with at least one tumor assessment post-treatment, more than half of the patients (8/14) discontinued due to extracranial disease progression and majority of the intracranial lesions kept stable.

## Discussion

Several ATP competitive TKIs against the HER2 protein have been approved for clinical use in recent years. These included the quinoline-based irreversible inhibitors neratinib and pyrotinib [19], and the quinazoline-based reversible inhibitors lapatinib and tucatinib [19]. HER2 belongs to the ErbB protein family, which includes the EGFR, HER2, HER3, and HER4. Although inhibition of wild-type EGFR is a known factor associated with diarrhea and rash observed in clinical practice, tucatinib was the only HER2 inhibitor specifically designed to avoid activity against the wild-type EGFR [8]. Another overlooked, but essential property in the design of HER2 inhibitors with clinical relevance is their ability to penetrate the BBB to treat patients with CNS metastases. Although the aforementioned clinically approved HER2 inhibitors have been used in the clinic with varying degrees of efficacy in patients with BM, there is still a need for additional HER2-selective inhibitors with more complete BBB penetration to further improve clinical efficacy in patients who have developed CNS metastases.

Although irreversible scaffolds dominated recent inhibitors designed for the ErbB family, selectivity against wild-type EGFR and avoiding being cell membrane transporter substrates (e.g., P-gp, BCRP) for brain penetration is challenging for those chemical scaffolds. The 4-([1,2,4]triazolo[1,5-a]pyridin-7-yloxy)-3-methylphenyl scaffold in the reversible HER2 inhibitor tucatinib provided a unique example for achieving the HER2 selectivity against wild-type EGFR as well as other kinases [20]. This N-hetero-biaryl occupied the ATP binding back pocket next to the gatekeeper residue Thr-798 and interacted with residue Ser-783 to enhance the binding potency with the HER2 protein.

Our expertise in designing ATP competitive brain-penetrant kinase inhibitors suggested that modification of compound’s substitution patterns occupying the solvent channel and/or the ribose-binding pocket spaces can profoundly affect their brain penetration ability, especially for quinazoline hinge binding motifs. In this disclosure, we provided another successful example in improving a clinically approved kinase inhibitor with a quinazoline scaffold to a true brain-penetrable compound without comprising its target potency and selectivity [21].

The brain is protected by several barriers, of which the most important is the BBB. The BBB is made up of endothelial cells forming the blood capillaries of the brain. These cells are tightly packed, meaning that the transport of molecules into the brain can occur only through transcellular passive diffusion or active uptake. The endothelial cells are also rich in efflux transporters, specifically P-gp, BCRP, and multidrug resistance proteins (MRPs), which are expressed on the luminal membrane and serve to pump molecules from the brain into the blood.

In the drug discovery phase, for the better treatment of CNS metastases, it is essential that compounds are well designed to have good brain exposure to ensure a therapeutic effect. In our screening paradigm, using cells expressing human P-gp and BCRP transporters to measure permeability and bi-directional efflux played an essential role in candidate selection. It was noted that preclinical in vivo rodent models might not truly reflect the brain penetrability of the compounds. The discrepancy observed may partly be due to the difference in transporter’s specificity among different species. As a result, it is essential to have suitable preclinical models and appropriate measures to confidently predict the likely brain exposure in human. DZD1516 crossed cells with moderate to high passive permeability and was not a substrate of P-gp and BCRP expressing at the BBB. DZD1516 also demonstrated favorable CNS penetration in rats and monkeys. This non-clinical evidence predicts effective CNS penetration of DZD1516 and DZ2678 in human.

For optimization of inhibitor structure, screening for an adequately positioned basic functional group into a quinazoline scaffold followed by fine-tuning the basicity of the compounds proved a viable strategy for optimizing candidates with good brain penetration properties. In this case, an oxygen-linked 3,3-difluoro-N-methylpiperidine moiety at the C5 position of the quinazoline hinges binding modified to the successful identification of DZD1516 as a true brain-penetrable candidate for clinical development.

DZD1516, with good BBB penetration, offers the potential to improve therapeutic outcomes in CNS settings as it can cross BBB and increase brain exposure with the potential to treat micro-metastatic lesions. Other HER2 inhibitors, including tucatinib, may have good intrinsic permeability [22–25]; however, they are substrates of BBB efflux transporters at various degrees, which affect their distribution into the brain at equilibrium. In contrast, DZD1516 and DZ2678 do not undergo active efflux via P-gp and BCRP in vitro and demonstrated similar unbound exposure between the CNS and plasma in rats and monkeys. Overall, non-clinical data support the favorable properties of DZD1516 crossing the BBB, delivering CNS exposure into the brain, and subsequently achieving pharmacological activity at sites of action. The BBB-penetrable property of DZD1516 was translated into better antitumor activity in the brain metastasis tumor xenograft models than other compounds.

As expected, clinical PK data further confirm effective CNS penetration of both DZD1516 and DZ2678 in human with *K*_p,uu,CSF_ close to 1. Moreover, free trough concentrations of DZD1516 and DZ2678 could cover in vitro pHER2 IC_50_ of BT474C1 cells with favorable PK properties, suggesting that 100 mg twice daily or above could achieve sustained blockage of pHER2 signaling pathways. An integrated approach, incorporating non-clinical PK/PD/efficacy analysis, clinical PK, safety, and tolerability data, allows for characterization of DZD1516 risk–benefit balance and supports dose finding in phase 2 trial in the target patient population.

This phase 1 study determined 250 mg BID as the MTD of DZD1516 monotherapy. DZD1516 was well tolerated with no ≥ grade 4 treatment-related adverse events or AEs leading to treatment discontinuation. The commonly reported AEs were headache, vomiting, and hemoglobin decreased. The majority of the AEs were grade 1 and were reversible. In comparison, vomiting was also reported in neratinib and tucatinib monotherapy studies with numerically higher incidences [26, 27]. Consistent with its high selectivity, no wild-type EGFR-related AEs have been reported during treatment with DZD1516 at MTD or lower doses. This compares favorably to the approximately 10% to 30% incidence of grade 3/4 diarrhea associated with lapatinib, neratinib, pyrotinib, and tucatinib at RP2Ds to treat metastatic breast cancer [8, 27–29].

The best efficacy is stable disease in the small patient population in this study. The possible explanation could be that the patients enrolled were heavily pretreated, especially with prior treatment of HER2 TKIs, which induced the resistance mechanism that could also be resistant to DZD1516. Currently, the HER2 TKIs are all approved in combination with other therapies for HER2+ breast cancer due to the add-on benefit. In this context, DZD1516 is expected to be more effective in combination with HER2 ADCs or antibodies, given the preclinically observed synergistic antitumor effect of DZD1516 in combination with HER2 ADCs in both extracranial and intracranial xenograft models.

In conclusion, DZD1516 is a full BBB-penetrant HER2 inhibitor and was well tolerated at doses ≤ 250 mg in patients with HER2+ metastatic breast cancer. Further clinical evaluation of DZD1516 in combination with HER2 ADCs in HER2+ metastatic breast cancer, especially in breast cancer with CNS metastases, is warranted.

## Supplementary Information

**Additional file 1**. Supplementary methods.

**Additional file 2**. **Table S1**: A brief SAR summary for the design of DZD1516. **Table S2**: In vitro inhibitory activityof DZD1516 against 12 kinases which were inhibited > 50% at 1 µM of DZD1516. **Table S3**: Summary of Single-Dose and Multiple-Dose Pharmacokinetics Parameters of DZD1516 and DZ2678 after Twice Daily Administration of DZD1516. **Table S4**: Listing of K_p,uu,CSF_ of DZD1516. **Table S5** Listing of K_p,uu,CSF_ of DZ2678.

**Additional file 3**. **Figure S1**: Enzymatic activity and cellular potency of DZD1516. A. Enzymatic activity of DZD1516 and its active metabolite DZ2678 on HER2 protein. The compound was pre-incubated with recombinant kinases at room temperature for 30 minutes. Then the reaction was initiated by adding 2 mM ATP and substrate peptide, where kinases could be phosphorylated in the reaction. After 60 minutes of incubation, the reaction was stopped by adding a detection reagent mix containing EDTA. B. The kinases with > 50% inhibition by 1 µM DZD1516 at Km ATP were plotted on the human kinome tree. Circle size is proportional to percentage inhibition. C. pHER2 IC_50_ of DZD1516 in HER2+ BT474C1or pEGFR IC_50_ of DZD1516 on A431 repressing wild-type EGFR. Cells were treated with a series of concentrations of DZD1516 for 4 hrs; then, pHER2 or pEGFR was measured with MSD SECTOR® Imager. A431 cell line was stimulated with 100 ng/ml of recombinant human EGF for 10 minutes after compound treatment before lysis. pEGFR: phosphorylated EGFR. Unpaired t test analysis *: P < 0.05. D. Antiproliferation activity GI_50_ of DZD1516 in BT474C1 or A431 cell lines. Cells were treated with a series of concentrations of DZD1516 for 72 hrs, and then the cell viability was analyzed using the CellTiter-Glo viability assay. Data were presented as the mean ± standard error of the mean. Unpaired t test analysis *: P < 0.05

**Additional file 4**. **Figure S2**: Representative image of H&E staining on brain tissues from LM mice model. The LM model was established by implanting tumor cells through cisterna magna under anesthesia following the same model development protocols as described in Supplementary method. Mice were killed xx days after intracisternal injection with tumor cells. Brains were excised and proceed for routine histological examination. H&E: hematoxylin and eosin. Histological evaluation at day 14 revealed leptomeningeal growth of multi-layered large polygonal cells with abundant cytoplasm, large nuclei and prominent nucleoli, and frequent mitoses figures. There was no invasion of the brain parenchyma.

**Additional file 5**. **Figure S3**: Body weight change of DZD1516 in combination with T-DM1 or T-Dxd in BT474C1-Luci Mono1 xenograft models. A. Body weight change of DZD1516 in combination with T-DM1 in the BM xenograft mice model. n = 9/group. B. Body weight change of DZD1516 in combination with T-DM1 in the SC xenograft mice model. n = 10/group. C. Body weight change of DZD1516 in combination with T-DXd in the BM xenograft mice model. D. Body weight change of DZD1516 in combination with T-DXd in the SC xenograft mice model. BID: twice daily; q2w: once every two weeks. qw: once weekly. BM: brain metastasis. SC: subcutaneous.

**Additional file 6**. **Figure S4**: Representative images of pHER2 IHC post-single-dose of DZD1516 in subcutaneous BT474C1-Luci Mono1 xenograft model. The mice were treated with a single dose of DZD1516 at 25 mg/kg, 50 mg/kg, and 150 mg/kg, respectively. The tumor tissues at different timepoints post-single-dose of DZD1516 were collected, and pHER2 expression in the tumor tissues was analyzed by IHC assay. Each time point had tumor tissues from three mice to detect the pHER2 signal. IHC: immunohistochemistry.

**Additional file 7**. **Figure S5**: Clinical activity of DZD1516 in heavily pre-treated breast cancer patients. Swimmer plot of tumor response over time. The study drug was administrated since C0D1. Treatment duration was presented since C1D1.

## Data Availability

All the data supporting the findings of this study are available within the article and its supplementary information files and from the corresponding authors upon reasonable request.
