# Supplementary material for: Preclinical and clinical activity of DZD1516, a full blood–brain barrier-penetrant, highly selective HER2 inhibitor
Source: Breast Cancer Res. 2023 Jul 6;25:81. doi: 10.1186/s13058-023-01679-4 (PMC10327353; doi:10.1186/s13058-023-01679-4)
Supplement: Supplementary file 1 — Additional file 1. Supplementary methods. [file 13058_2023_1679_MOESM1_ESM.docx]

### **Supplementary methods**

***Cell lines***

BT474 and A431 were purchased from the American Type Culture collection. BT474 cells were cultured in DMEM medium supplemented with 10% newborn calf serum (NBCS; Gibco) and 1 mM OAA (Sigma). A431 cells were cultured in DMEM (Gibco) with 10% FBS. All cells were maintained and propagated as monolayer cultures in a humidified incubator with 5% CO_2_ at 37°C.

***Establishment of BT474C1-Luci Mono1 cell clone***

In order to monitor the tumour growth in brain by measuring the bioluminescence signals in tumour cells using the imaging system, the stable cell clone BT474C1-Luci Mono1 was generated by transfecting human breast cancer cell BT474 with pGL4.50[luc2/CMV/Hygro] vector which contains luciferase gene using lipofectamine LTX (Invitrogen™, USA). The clone was selected with 0.5 μg/ml puromycin by serial dilution and confirmed HER2 positive. This clone was used as HER2+ cell model in this article.

***In vitro enzymatic assay***

Recombinant HER2 was purchased from Thermo Fisher (Cat. # PR4918A). The inhibition potency of compounds against HER2 enzyme was assessed using Lance Ultra Kinase Assay.

In brief, recombinant kinases were pre-incubated in the presence or absence of the compound at room temperature for 30 minutes. The reaction was initiated by the addition of the ATP and substrate peptide. After 60 minutes of incubation, the reaction was stopped by the addition of the detection reagent mix containing EDTA. The fluorescence was measured at 615 nm and 665 nm, respectively, with excitation wavelength at 320 nm. The calculated signal ratio of 665 nm/615 nm is proportional to the kinase activity. The concentration of the compound producing 50% inhibition of the respective kinase (IC_50_) was calculated using a four-parameter logistic fit with XLfit.

**Kinase panel assay**

*In vitro* profiling of the 121-recombinant kinase panel was performed at Eurofins UK Ltd (Dundee, UK). In brief, recombinant kinases were incubated within an appropriate buffer containing peptide substrate and radiolabelled γ-^33^P-ATP together with the presence or absence of the required compound concentration. The reaction was initiated by adding ATP/Mg^2+^ mix. After incubation for 40 minutes at room temperature, the reaction was stopped by adding 3% phosphoric acid solution. A portion of the reaction mix was spotted onto P30 filtermat to trap peptide and washed three times for 5 minutes with phosphoric acid to remove non-specific γ-^33^P-ATP. The substrate phosphorylation was then measured by scintillation counting, which determined the level of kinase activity inhibition compared to control reactions. Percentage inhibition data for each kinase was mapped to the kinome tree ([www.kinhub.org](http://www.kinhub.org)).

***pHER2 or pEGFR MSD assay***

A431 and BT474C1-Luci Mono1 cells were seeded in 96-well plates at 20,000 cells/well in DMEM medium containing 1% FBS. After overnight incubation, all cells were treated with compounds at a series of concentrations for 4 hrs. For A431 cell line, an extra stimulation with recombinant human EGF (100 ng/ml for 10 minutes) is applied to activate wild-type EGFR following compound treatment. pEGFR^Y1068^ and total EGFR levels were measured by an electro chemiluminescent method (MULTI-SPOT®96 4-Spot HB Prototype EGFR Triplex ANALYTES: pEGFR (Y1068), Total EGFR; MESO SCALE DISCOVERY, Cat# N45ZB-1) with MSD SECTOR® Imager, which outputs the ratio of pEGFR/total EGFR for each well. pHER2^Y1248^ and total HER2 levels were measured similarly using pHER2 (Y1248) Assay Whole Cell Lysate Kit (MESO SCALE DISCOVERY, Cat# K151CLD-3). The percentage of inhibition was calculated as: % Inhibition = 100 x [1 - (ratio of the sample well - ratio of Min ctrl well)/ (ratio of Max ctrl well - ratio of Min ctrl well)]. The concentration of the compound producing 50% inhibition of EGFR or HER2 phosphorylation was calculated in best-fit curves with Prism GraphPad (RRID:SCR_002798, GraphPad Software Inc).

***Cell proliferation assay***

BT474C1-Luci Mono1 cells were seeded in 384-well plates at 1500 cells/well in DMEM medium containing 10% FBS and 1 mM Oxaloacetic acid (OAA). A431 cells were seeded in 384-well plates at 1,000 cells/well in DMEM medium containing 10% FBS. After overnight incubation, cells were treated with compounds at various concentrations for 3 days. Then cell viability was measured using a CellTiter-Glo Luminescent Cell Viability Assay from Promega Corp. according to the manufacturer's instructions.

***Mice***

6 to 8-week-old specific-pathogen-free immunodeficient NU/NU Mice were purchased from Beijing Vital River Laboratory Animal Technology Co., Ltd. All the animal studies were approved by IACUC and conducted in compliance with standard and local regulatory requirements of Dizal Pharmaceutical.

***Establishment of BM and LM models***

The BM model was established by intracerebral (ICB) injection of 4 μl of cell suspension containing 3 × 10^5^ cells into each mouse following similar procedures published (30). In brieft, 17β- ESTRADIOL (90-day release 0.36 mg/pellet) were injected subcutaneously 24 hrs before cell inoculation. Under anesthesia, a sagittal incision (approximately 1 cm long) was made over the parieto-occipital bone using a sterile scalpel. Then a hole was punctured on the skull at 2.5 mm to the right of the bregma and 1 mm anterior to the coronal suture using a sterile 25-gauge sharp drill. The syringe perpendicular was placed to the skull through the hole and reach 3 mm deep below the skull surface and BT474C1-Luci Mono1 cell suspension was injected with an infusion pump. Afterwards, the sterile bone gel was applied to the hole and the wound was closed by using wound clips or silk sutures. The bioluminescent signals of the implanted cells were measured by the Xenogen IVIS spectrum imaging system (PerkinElmer, USA) to monitor tumor growth. When signals reached > 1 × 10^7^ photon/s, animals were randomly assigned to different treatment groups.

The LM model was generated by implanting 10 μl of cell suspension containing 1 × 10^4^ BT474C1-Luci Mono1 cells through cisterna magna into each mouse under anaesthesia following similar procedures as described in the literature (31). 17β- ESTRADIOL (90-day release 0.36 mg/pellet) were injected subcutaneously 24 hrs before cell inoculation. Under anaesthesia, mice were placed in the prone position with their neck draped over a cylinder. The head was immobilized with the thumb and mid-finger, with the index finger palpating the space between the occiput and C1. BT474c1-Luci Mono1 cell suspension at 10 µl containing 0.01 million cells were injected intracisternally. When the bioluminescent signal reached > 5 × 10^6^ photon/s, animals were randomized for treatment. Model characterization has been done by assessing tumor cells in brain tissues with histological haemotoxylin and eosin (H&E) staining (Supplementary Figure S2).

In both BM and LM models, the day of randomization was defined as Day 0 and the treatment was started one day after randomization. The bioluminescence signals and body weight of the mice were measured once weekly. Relative bioluminescence intensity on a specific day was calculated by dividing the bioluminescence intensity on that day by the bioluminescence intensity on Day 0. The tumour growth inhibition (TGI) was calculated by formula: TGI (%) = (1-(relative bioluminescence intensity of treated group)/(relative bioluminescence intensity of control group)) x 100.***Establishment of SC model***

Tumor cell suspension at 5 × 10^6^ were injected subcutaneously in a total volume of 0.2 ml (1:1 with ECM Gel) into each mouse. 17β-ESTRADIOL (90-day release, 0.36 mg/pellet) were implanted 24 hrs before cells were injected subcutaneously. Tumor nodules were measured in two dimensions with caliper and the tumor volume was calculated using the following formula: tumor volume = (length × width^2^) × 0.5. Tumor-bearing mice were randomized into different treatment groups when the mean tumor volume reached around 150 mm^3^.

***Assessment of passive permeability and efflux ratio of P-gp and BCRP transporters***

Passive permeability (Papp) characterizes the rate across the cell membrane due to passive diffusion. A high permeability enables the rapid establishment of equilibrium among plasma, CSF, and brain compartments. Except for passive permeability, sufficient unbound drug concentration in the brain is governed by efflux transporters, including P-gp (*MDR1, ABCB1*) and BCRP, at BBB. Efflux ratio (ER) reflects the liability of a compound to be pumped out of the brain by BBB efflux transporters, which is indicated by the distribution disequilibrium between the plasma and the brain compartments.

DZD1516 and DZ2678 permeability was assessed in a bi-directional assay in MDCKII-MDR1 and mock MDCK II cells at 5 μM. The adjusted efflux ratio was used to determine if DZD1516/DZ2678 underwent active efflux. Verapamil, a P-glycoprotein (P-gp) inhibitor, was used to identify if any active transport processes were mediated through P-gp. The apical-to-basolateral (A-B) and basolateral-to-apical (B-A) transport of DZD1516/DZ2678 in HBSS (25 mM HEPES, pH 7.4) with 0.1% BSA was measured across MDCKII-MDR1 cell monolayers in the absence and presence of the P-gp inhibitor, verapamil (100 μM). The apical-to-basolateral (A-B) and basolateral-to-apical (B-A) transport of DZD1516/DZ2678 in HBSS (25 mM HEPES, pH 7.4) with 0.1% BSA was also measured across MDCKII cell monolayers in the absence and presence of the P-gp inhibitor, verapamil. Incubations were performed at approximately 37°C for 120 min, with the functionality of the test system being confirmed using 5 μM digoxin as a positive control substrate. Transport of 5 μM DZD1516/DZ2678 and control compound were determined by quantifying substrate concentration in the incubation medium of the donor compartment at the beginning of the incubation period and both donor and receiver compartments at the end of the incubation period. The data was used to calculate the apparent permeability (Papp). All incubations were performed in triplicate, and the integrity of the cell monolayers was confirmed using the marker Lucifer yellow.

Again, DZD1516 and DZ2678 permeability were evaluated in a bi-directional assay in Caco-2 cells at 5 μM. The efflux ratio of DZD1516 and DZ2678, with or without novobiocin (a selective BCRP inhibitor), determined whether DZD1516 or DZ2678 was transported via BCRP in Caco-2 cells. The apical-to-basolateral (A-B) and basolateral-to-apical (B‑A) transport of DZD1516/DZ2678 in HBSS (25 mM HEPES, pH 7.4) with 0.1% BSA was measured across Caco-2 cell monolayers in the absence and presence of BCRP inhibitor, novobiocin (30 μM). Incubations were performed at approximately 37°C for 120 min, with the functionality of the test system being confirmed using 5 μM rosuvastatin as a positive control substrate. Transport of 5 μM DZD1516/DZ2678 and control compound was determined by quantifying substrate concentration in the incubation medium of the donor compartment at the beginning of the incubation period and both donor and receiver compartments at the end of the incubation period. The data was used to calculate the apparent permeability (P_app_). All incubations were performed in triplicate, and the integrity of the cell monolayers was confirmed using the marker Lucifer yellow.

**Apparent permeability (P_app_,cm/s × 10^-6^)** can be calculated for drug transport assays using the following equation:

*$P_{app}=\frac{V_{A}}{Area\times time}\times\frac{\left[drug\right]_{receiver}}{\left[drug\right]_{initial,donor}}$*

Where: V_A_ is the volume (in mL) in the receiver well (0.3 mL for Ap→Bl flux and 0.1mL for Bl→Ap flux)
Area is the surface area of the membrane (0.143 cm^2^ for HTS Transwell-96 Well Permeable Supports)
Time is the total transport time in seconds

**Efflux ratio** and **Net flux ratio** can be determined using the following equation:

$$\text{Net Flux Ratio}=\frac{\text{Efllux Ratio}_{\text{(MDCK-MDR1)}}}{\text{Efflux Ratio}_{\text{(MDCK)}}}$$

Where: P_app (B-A)_ indicates the apparent permeability coefficient in basolateral to apical direction
P_app (A-B)_ indicates the apparent permeability coefficient in the apical to basolateral direction.

**Mass balance (recovery %)** can be determined using the following equation:

*$\mathrm{Recov}ery\%=\frac{\left[drug\right]_{receiver}\times V_{A}+\left[drug\right]_{donor}\times V_{D}}{\left[drug\right]_{initial,donor}\times V_{D}}\times100$*

Where: V_A_ is the volume (in mL) in the receiver well
V_D_ is the volume (in mL) in the donor well

**Lucifer yellow leakage** of the monolayer can be calculated using the following equation:

Where: I_acceptor_ is the fluorescence intensity in the acceptor well (0.3 mL)
I_donor_ is the fluorescence intensity in the donor well (0.1 mL) and expressed as % leakage.
Lucifer yellow percentage amount transported values should be less than 1.5 %

***Fraction unbound of DZD1516/DZ2678 in plasma and brain tissue***

Fraction unbound (f_u_) in plasma and brain using equilibrium dialysis tissue converts total drug concentration, obtained from *in vivo* CNS-PK testing, to unbound concentration in plasma or brain.

The *in vitro* binding of DZD1516 and DZ2678 to plasma proteins was assessed among untreated mouse, rat, dog, monkey, and human samples over the range 0.1 - 100 μM by rapid equilibrium dialysis (RED) device over 18-hr incubation. Again, the *in vitro* binding of DZD1516 and DZ2678 to rat brain tissues was assessed using the untreated brain homogenate in the range of 0.1 - 10 μM by RED device over 16- or 18-hr incubation time.

The stability assessment and determination of the time to equilibrate DZ2678 at oneμmol/L and tenμmol/L were performed at 0, 5, 6, 16, 18, and 20 hrs in human plasma and PBS (phosphate buffer, 100 mM, pH 7.4). According to the results, the plasma protein binding assay was performed with 18 hrs of incubation in plasma.

The stability assessment and determination of the time to equilibration of DZ2678 at one μmol/L were performed at 0, 5, 6, 16, 18, and 20 hrs in PBS (phosphate buffer, 100 mM, pH 7.4), and rat brain homogenate (rat brain tissue (g): PBS (mL) =1:4 and 1:10). According to the results, the binding assay was performed with 18 hrss of incubation in rat brain homogenate (1:10).

**Protein binding**

All calculations are carried out using Microsoft Excel.

Calculate the percentage of unbound, percentage of bound and recovery as follows:

% Unbound = (Conc. _buffer chamber_ / Conc. _protein chamber_) × 100

% Bound = 100% - % Unbound

% Recovery = (Conc. _buffer chamber_ × 500 + Conc. _protein chamber_ × 300) / (Conc. _Total sample_× 300) × 100

**Plasma stability**

All calculations are carried out using Microsoft Excel.

Calculate the remaining percentage (%) at each time point as follows:

Remaining% = Conc. _time point_ / Conc. _0hr_ × 100

***In vivo CNS-PK studies to evaluate CNS penetration of DZD1516/DZ2678***

The ability of DZD1516 and DZ2678 to cross the BBB in female Wistar Han rat and female Cynomolgus monkey was studied by assessing the free brain-to-plasma ratio (K_p,uu,brain_) and CSF-to-free plasma ratio (K_p,uu,CSF_). Total brain-to-blood ratio (K_p,brain_) and CSF-to-blood ratio (K_p,CSF)_ were measured from six time points after oral administration. Free fractions of DZD1516 and DZ2678 in the biological matrix were determined by *in vitro* plasma and brain binding assay.

K_p,brain_= AUC_brain_/AUC_blood_. K_p,CSF_ was the average of the CSF-to-blood ratios at the evaluated time points. K_p,uu,brain_= K_p,brain_× fu,brain/fu,blood; K_puu,CSF_= K_p,CSF_/fu,blood.

In total, twenty-one female Wistar Han rats were administered with a single PO dose of DZD1516 at 30 mg/kg. The PO dosing formulation was a suspension in 0.5% HPMC E5 in water (w/v). Plasma, brain, and CSF samples were collected at 0.5, 1, 2, 4, 8, 24, and 30 hrs post-dose. The concentrations of DZD1516 and metabolite DZ2678 in plasma, brain, and CSF samples from the rats, as well as in plasma and CSF samples from the monkeys, were determined by the validated LC-MS/MS method.

Three female Cynomolgus monkeys were administered with a single PO dose of DZD1516 at 30 mg/kg. The PO dosing formulation was a suspension in 0.5% HPMC E5 in water (w/v). Plasma and CSF samples were collected at 0.5, 1, 2, 4, 8, and 24 hrs post-dose. The concentrations of DZD1516 and metabolite DZ2678 in monkey plasma and CSF were determined by the validated LC-MS/MS method.

Standards were prepared by spiking blank rat plasma, monkey plasma, rat brain homogenate, and artificial CSF covering 3.0 to 3000 ng/mL, 1.0 to 1000 ng/mL, 0.5 to 500 ng/Ml, and 0.2 to 200 ng/mL, respectively. Homogenized brain tissue, plasma, and CSF samples were precipitated by adding an appropriate volume of cold acetonitrile containing internal standard ([D_6_]-DZD1516 and [D_6_]-DZ2678, 0.5 ng/mL). After 2 min vortex and 5 min centrifugation at 14,000 rpm, the supernatant was analyzed by LC/MS/MS (Triple Quad 6500+, Applied Biosystems, Foster City) and Shimadzu LC-30AD pump, SIL-30AC autosampler, CTO-20A column oven, and CBM-20A controller. Two sets of standard curves were run at the beginning and end of each batch from blood sample analysis. For brain and CSF samples, one standard curve was analyzed along with test samples.

Pharmacokinetic parameters were estimated using WinNonlin® Professional 6.4 with a non-compartmental analysis model.

***Immunohistochemical (IHC) staining for pHER2 in xenograft tumor tissues***

The subcutaneous xenograft tissues were collected from mice after a single dose of vehicle or DZD1516. Samples were snap-frozen for analysis. Tissues from animals were analyzed at each time point IHC was performed on 5 μm frozen sections using a Ventana discovery XT automation (Roche) for pHER2 (Catalog #) IHC staining. The stained IHC slides were firstly reviewed and interpreted by a qualified pathologist and then quantified by the Imagescope software of Aperio system. The “H” score quantified by Aperio system will be corrected by a pathologist. Statistical analysis was performed by t-test.

***Clinical Study design and treatment***

DZD1516 was dosed orally as a tablet formulation at 25 mg or 100 mg unit strengths.

Eligible patients received single oral dosing of DZD1516 on Cycle 0 Day 1 (C0D1), followed by a 7-day washout. Twice daily oral dosing (bid) of DZD1516 was commenced from C1D1 in a continuous 21-day cycle until disease progression, unacceptable toxicity, or withdrawal of consent. All doses were taken under fed conditions (within 30 minutes after a meal).

Planned dosing consisted of 25, 50, 100, 200, 300, and 400 mg. Each dose escalation cohort will comprise a minimum of three and a maximum of six evaluable patients (except only one patient in the starting dose cohort). The maximum tolerated dose of DZD1516 was defined according to safety data and the Bayesian optimal interval (BOIN) design. Dose escalation/de-escalation rule with target toxicity rate for the MTD is 0.30 with interval of (0.20, 0.33).

DLTs were any adverse event (AE) not clearly attributable to the patient’s disease, including ≥ grade 4 hematological toxicities present for ≥ 4 days, grade 3 thrombocytopenia with bleeding, or grade 4 thrombocytopenia lasting for > 5 days, ≥ grade 3 non-hematological toxicities that is dose-limiting in the judgment of the investigators. Any other toxicity that is greater than that at baseline, is clinically significant or unacceptable, and is judged to be a DLT by the SRC. Exceptions included alopecia of any grade and isolated grade 3 laboratory change without clinical sequelae or clinical significance.

***Patient population***

Patients were eligible for enrollment if they were ≥ 18 years old, had a histological or cytologic diagnosis of MBC, and were HER2 positive (immunochemistry 3+, or immunochemistry 2+ confirmed by fluorescent in situ hybridization). Patients with CNS metastases are eligible if there is no indication for urgent neurosurgical intervention. Additional eligibility criteria included Eastern Cooperative Oncology Group (ECOG) performance status 0-1 (for patients with LM, ECOG of 2 is acceptable),), adequate organ function and bone marrow reserve, QTc interval ≤ 450 ms, and a left ventricular ejection fraction ≥ 55% assessed by ECHO or MUGA. There were no limitations on the number of prior lines of systemic therapy; however, patients were required to have documented disease progression on the most recent disease evaluation. Exclusion criteria included concurrent systemic cancer therapy or other investigational agents, uncontrolled illness or active infection, history of significant cardiac disease, cardiac risk factors or uncontrolled arrhythmias, and women who are pregnant or breastfeeding.

***Safety analysis***

Safety evaluations were conducted at screening, on Day 1, 8, and 15 of Cycle 1, on Day 1 of each subsequent treatment cycle, at the end-of-treatment visit, and clinically indicated. Safety assessments included clinical laboratory measurements (hematology, chemistry, coagulation, and urinalysis), electrocardiogram (ECG), Echocardiogram/MUGA scan, ECOG performance status, vital signs, and physical examinations.

The AE grading was assessed according to the NCI-Common Terminology Criteria for Adverse Events (CTCAE) (version 5.0) and was monitored until 28 days after the last dose.

***Efficacy analysis***

Tumor imaging assessments were conducted every six weeks during the first 24 weeks (relative to C1D1) and every nine weeks thereafter per investigator review according to modified RECIST v1.1 until progressive disease, starting a new anti-cancer therapy or withdrawal of informed consent. Patients who achieved an objective response had confirmatory scans at least four weeks apart.

The modified RECIST 1.1 used in this study includes both CNS assessment and extracranial assessment. A maximum of five measurable extracranial lesions, with a maximum of two lesions per organ, were selected in patients with extracranial disease. In addition, a maximum of five measurable intracranial lesions were selected in patients with intracranial disease. The RECIST (version 1.1) guidelines for measurable, non-measurable, target, and non-target lesions and the objective tumor response criteria (complete response, partial response, stable disease, or progression of disease) will be used for the assessment of tumors. An overall response will be derived based on extracranial and intracranial response as described in the below table.

| Extracranial lesion visit response | CNS lesion visit response | Overall response |
| --- | --- | --- |
| CR | CR | CR |
| CR | NA | CR |
| NA | CR | CR |
| CR | PR or SD | PR |
| CR | NE | PR |
| PR | CR/PR/SD or NE | PR |
| SD | CR/PR/SD or NE | SD |
| NA | PR/SD | SD |
| NE | Non PD or NE | NE |
| NA | NE | NE |
| PD | Any | PD |
| Any | PD | PD |

For patients without CNS metastases, only extracranial response will be assessed. And vice versa.

***Bioanalysis of plasma samples and pharmacokinetic assessments***

Blood samples for PK analyses of DZD1516 and DZ2678 were collected on C0D1 and C1D15.

Plasma concentrations of analytes evaluated in this study were determined using validated bioanalytical methods at Labcorp Madison lab (Madison, WI, USA) and Labcorp Shanghai lab (Shanghai, China). Concentrations of analytes in human plasma containing K2EDTA as an anticoagulant was determined using plasma precipitation, followed by analysis using high-performance LC-MS/MS detection. Two bioanalytical methods used 4 mL of plasma and were linear over the range of 5.00 (the lower limit of quantification) to 5000 ng/mL for DZD1516 and 2.00 (the lower limit of quantification) to 2000 ng/mL for DZ2678 (Labcorp Shanghai lab), 1.96 (the lower limit of quantification) to 1960 ng/mL for DZ2678 (Labcorp Madison lab). The analysis runs for all analytes were determined to have a high degree of reproducibility and acceptably low inter-run carryover, which was considered acceptable for PK quantification. The plasma samples were stored at –70°C until analysis. PK analyses were performed by using Phoenix WinNonLin (Certara USA, Inc., Version 8.1 or higher).

CSF samples for PK analyses of DZD1516 and its metabolite DZ2678 were collected from patients with BM (C1D15) and LM (C1D15 and C3D1) at steady state. CSF samples were assayed for DZD1516 and its metabolite DZ2678 using validated high-performance liquid chromatography-tandem mass spectrometry (HPLC-MS/MS) methods. The bioanalytical method used 0.3 mL of CSF and was linear over the range of 0.500 (the lower limit of quantification) to 200 ng/mL for DZD1516 and 0.250 (the lower limit of quantification) to 100 ng/mL for DZ2678, K_p,CSF_ and K_p,uu,CSF_ will be calculated.

**Statistical analysis**

The comparison of bioluminescent signals in the BM and LM models and tumor volume in the SC model in different treatment groups was performed by applying two-way ANOVA. All analyses were performed using GraphPad Prism software. A *p* value of < 0.05 was considered statistically significant.
