# Supplementary material for: Preclinical and clinical activity of DZD1516, a full blood–brain barrier-penetrant, highly selective HER2 inhibitor
Source: Breast Cancer Res. 2023 Jul 6;25:81. doi: 10.1186/s13058-023-01679-4 (PMC10327353; doi:10.1186/s13058-023-01679-4)
Supplement: Supplementary file 2 — Additional file 2. Table S1: A brief SAR summary for the design of DZD1516. Table S2: In vitro inhibitory activityof DZD1516 against 12 kinases which were inhibited > 50% at 1 µM of DZD1516. Table S3: Summary of Single-Dose and Multiple-Dose Pharmacokinetics Parameters of DZD1516 and DZ2678 after Twice Daily Administration of DZD1516. Table S4: Listing of Kp,uu,CSF of DZD1516. Table S5 Listing of Kp,uu,CSF of DZ2678. [file 13058_2023_1679_MOESM2_ESM.docx]

**Supplementary data – table and text**

**Supplementary Table S1. A brief SAR summary for the design of DZD1516**

| Reference | | | | BT474C1 | | A431 | Selected *in vitro* DMPK assays | | | |
| --- | --- | --- | --- | --- | --- | --- | --- | --- | --- | --- |
|  |  |  |  | pHER2  IC_50_ (nM) | GI_50_ (nM) | pEGFR IC_50_ (nM) | Efflux ratio | | | Human Heps Clint [(µL/min)/ (10^6^ cells)] |
|  |  |  |  |  |  |  | P-gp  @1 µM | BCRP  @1 µM | P-gp-BCRP  @1 µM |  |
| Neratinib  (DZ’2679) |  | | | 0.71 | 1.3 | 16 | 17.7 | 4.5 | >9.8 | 7.7 |
| Pyrotinib  (DZ’0745) |  | | | 0.66 | 0.7 | 25 |  |  | >16.6 |  |
| Lapatinib  (DZ’2680) |  | | | 4.8 | 13 | 45 | 15 |  | >4.1 |  |
| Tucatinib  (DZ’2681) |  | | | 1.7 | 10 | 2767 | 6.2 |  | 64 | 12.5 |
|  | R_1_ = | R_2_ = | R_3_ = |  |  |  |  |  |  |  |
| Cpd-1  (AZ13649129) | H | H |  | 18 | 91 | >10000 | 1.5 |  |  |  |
| Cpd-2  (AZ13696963) | H | H |  | 5.8 | 4.1 | >10000 | 0.77 | 0.54 |  | 218 |
| Cpd-3  (AZ13696962) | H | H |  | 11 | 25 |  | 0.84 |  |  |  |
| Cpd-4 (AZ13705672, DZ’1736)  (R)-enantiomer | -OMe | H |  | 44 | 67 |  |  |  |  | 20.1 |
| Cpd-5 (DZD1516)  (S)-enantiomer | -OMe | H |  | 4.4 | 20 | 1455 | 1.6 | 1.3 | 1.8 | 12.0 |
| Cpd-6  (DZ2678) | -OMe | H |  | 3.5 | 13 | 740 | 2.8 |  | 6.5 | 7.7 |

GI_50_: half-maximal growth inhibitory concentration

**Supplementary Table S2. *In vitro* inhibitory activity (IC_50_, nM) of DZD1516 against 12 kinases which were inhibited > 50% at 1 µM of DZD1516**

| **Kinase (Human)** | **IC_50_** |
| --- | --- |
| HER2(h) | 16 |
| ErbB4(h) | 52 |
| EGFR(h) | 92 |
| Lck(h) activated | 180 |
| EGFR(T790M,L858R)(h) | 244 |
| Lck(h) | 433 |
| c-RAF(h) | 1037 |
| Fms(h) | 1070 |
| Met(h) | 1093 |
| TrkA(h) | 1291 |
| Lyn(h) | 2301 |
| Blk(h) | 2667 |

**Supplementary Table S3** Summary of Single-Dose and Multiple-Dose Pharmacokinetics Parameters of DZD1516 and DZ2678 after Twice Daily Administration of DZD1516

|  |  |  | 250 mg BID | |
| --- | --- | --- | --- | --- |
|  | **Parameters** | **Statistics** | **DZD1516** | **DZ2678** |
| Day 1 | N |  | 5 | 5 |
|  | AUC_0-10_ (hr*ng/mL) | Geometric Mean [CV%] | 6155[24.30] | 3400 [51.89] |
|  | AUC_0-72_ (hr*ng/mL) | Geometric Mean [CV%] | 13180[35.88] | 7146 [48.56] |
|  | C_max_ (ng/mL) | Geometric Mean [CV%] | 1329[20.65] | 738.0 [61.14] |
|  | t_max_ (hr) | Median (Min, Max) | 2.850(0.983,6.00) | 5.867 (1.97,7.97) |
|  | t_1/2_ (hr) | Mean (SD) | 19.78(12.20) | 13.36 (4.503)^a^ |
| Day 15 | N |  | 5 | 5 |
|  | AUC_0-10_ (hr*ng/mL) | Geometric Mean [CV%] | 7555[45.37] | 4401 [55.16] |
|  | AUC_tau_ (hr*ng/mL) | Geometric Mean [CV%] | 8343[46.40] | NA |
|  | C_ss,max_ (ng/mL) | Geometric Mean [CV%] | 1410[44.64] | 724.0 [44.98] |
|  | C_ss,min_ (ng/mL) | Geometric Mean [CV%] | 442.8[50.12] | 237.1 [57.36] |
|  | t_ss,max_ (hr) | Median (Min, Max) | 1.483(0.533,2.47) | 2.383 (0.000,4.00) |
|  | Rac (AUC) | Mean (SD) | 1.316(0.5472) | NA |
|  | MR_AUC_ | Mean (SD) | NA | 0.5970 (0.1106) |

Note: Rac (AUC) was estimated by the following equation AUC(C1D15)/AUC(C0); MR_AUC_ was estimated by the following equation DZ2678(AUC_0-10_)/DZD1516(AUC_0-10_); N = Number of participants in the analysis set for each treatment group; ^a^ : Number of observations is 4;

**Supplementary Table S4** Listing of K_p,uu,CSF_ of DZD1516

| **Dose** | **Subject** | **CSF conc (nmol/L)** | **Free plasma conc (nmol/L)** | **K_p,uu,CSF_** |
| --- | --- | --- | --- | --- |
| 50 mg | subject 1 | 4.89 | 5.35 | 0.915 |
| 50 mg | subject 2 | 10.2 | 6.28 | 1.62 |
| 100 mg | subject 3 | 39.3 | 13.4 | 2.94 |
| 100 mg | subject 4 | 15.5 | 4.95 | 3.13 |
| 100 mg | subject 5 | 12.7 | 4.84 | 2.63 |
| 200 mg | subject 6 | 18.3 | 13.9 | 1.31 |
| 250 mg | subject 7 | 27.9 | 24.0 | 1.16 |
| 250 mg | subject 8 | 54.2 | 17.4 | 3.13 |

**Supplementary Table S5** Listing of K_p,uu,CSF_ of DZ2678

| **Dose** | **Subject ID** | **CSF conc (nmol/L)** | **Free plasma conc (nmol/L)** | **K_p,uu,CSF_** |
| --- | --- | --- | --- | --- |
| 50 mg | subject 1 | 0.549 | 2.52 | 0.218 |
| 50 mg | subject 2 | 1.12 | 1.47 | 0.765 |
| 100 mg | subject 3 | 3.15 | 7.23 | 0.436 |
| 100 mg | subject 4 | 2.10 | 1.31 | 1.60 |
| 100 mg | subject 5 | 1.65 | 1.66 | 0.995 |
| 200 mg | subject 6 | 3.95 | 7.20 | 0.549 |
| 250 mg | subject 7 | 3.88 | 9.15 | 0.424 |
| 250 mg | subject 8 | 9.13 | 8.58 | 1.06 |
