# Supplementary figures and images for: Preclinical and clinical activity of DZD1516, a full blood–brain barrier-penetrant, highly selective HER2 inhibitor
Source: Breast Cancer Res. 2023 Jul 6;25:81. doi: 10.1186/s13058-023-01679-4 (PMC10327353; doi:10.1186/s13058-023-01679-4)

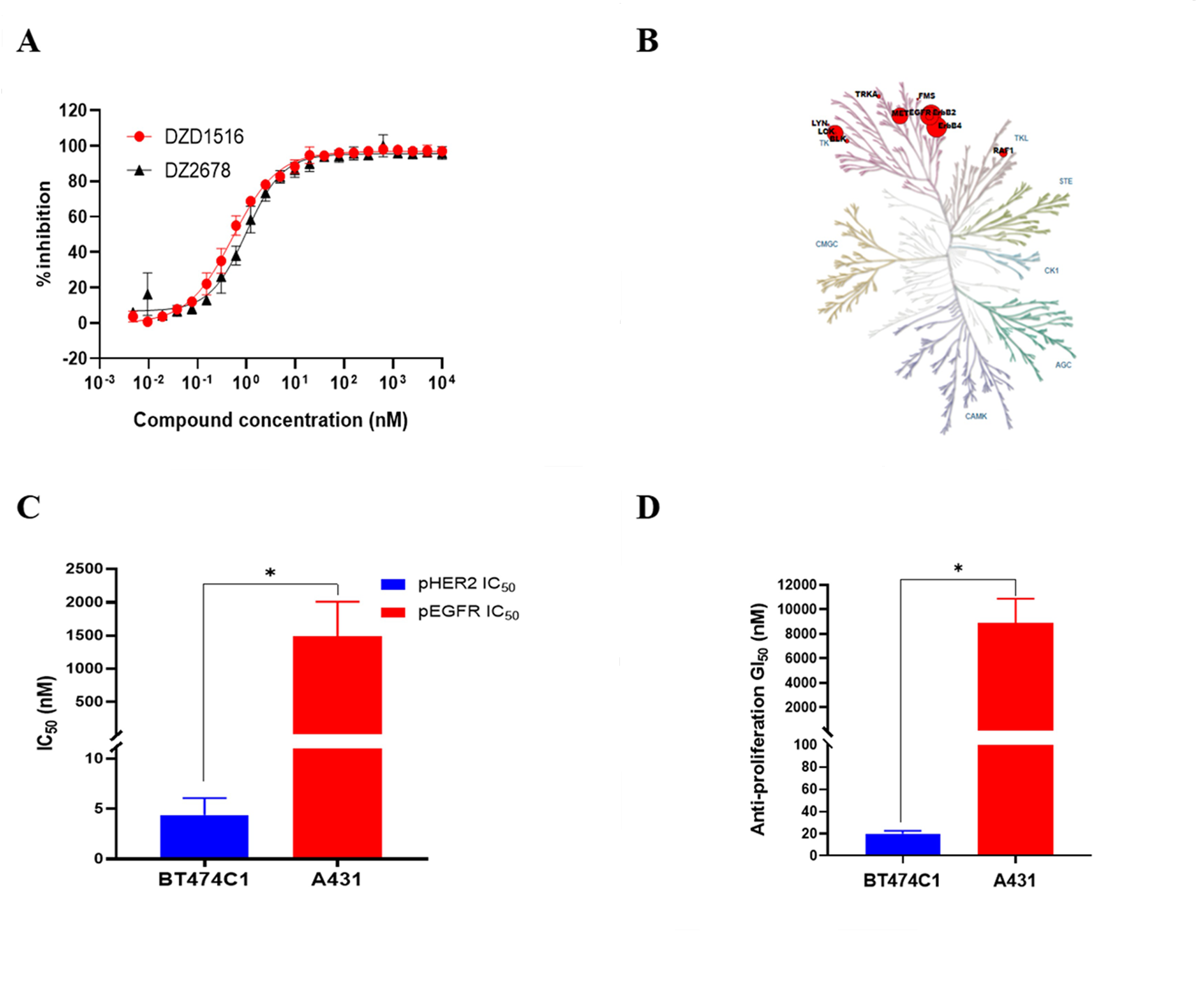

Supplement: Supplementary file 3 — Additional file 3. Figure S1: Enzymatic activity and cellular potency of DZD1516. A. Enzymatic activity of DZD1516 and its active metabolite DZ2678 on HER2 protein. The compound was pre-incubated with recombinant kinases at room temperature for 30 minutes. Then the reaction was initiated by adding 2 mM ATP and substrate peptide, where kinases could be phosphorylated in the reaction. After 60 minutes of incubation, the reaction was stopped by adding a detection reagent mix containing EDTA. B. The kinases with > 50% inhibition by 1 µM DZD1516 at Km ATP were plotted on the human kinome tree. Circle size is proportional to percentage inhibition. C. pHER2 IC50 of DZD1516 in HER2+ BT474C1or pEGFR IC50 of DZD1516 on A431 repressing wild-type EGFR. Cells were treated with a series of concentrations of DZD1516 for 4 hrs; then, pHER2 or pEGFR was measured with MSD SECTOR® Imager. A431 cell line was stimulated with 100 ng/ml of recombinant human EGF for 10 minutes after compound treatment before lysis. pEGFR: phosphorylated EGFR. Unpaired t test analysis *: P < 0.05. D. Antiproliferation activity GI50 of DZD1516 in BT474C1 or A431 cell lines. Cells were treated with a series of concentrations of DZD1516 for 72 hrs, and then the cell viability was analyzed using the CellTiter-Glo viability assay. Data were presented as the mean ± standard error of the mean. Unpaired t test analysis *: P < 0.05 [file 13058_2023_1679_MOESM3_ESM.tif]

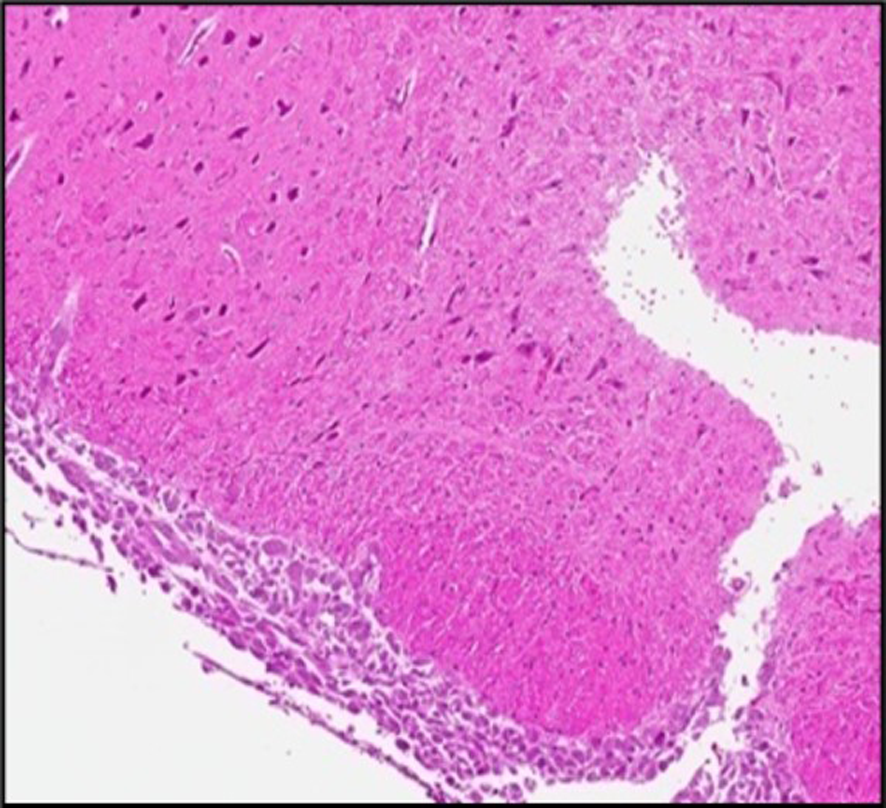

Supplement: Supplementary file 4 — Additional file 4. Figure S2: Representative image of H&E staining on brain tissues from LM mice model. The LM model was established by implanting tumor cells through cisterna magna under anesthesia following the same model development protocols as described in Supplementary method. Mice were killed xx days after intracisternal injection with tumor cells. Brains were excised and proceed for routine histological examination. H&E: hematoxylin and eosin. Histological evaluation at day 14 revealed leptomeningeal growth of multi-layered large polygonal cells with abundant cytoplasm, large nuclei and prominent nucleoli, and frequent mitoses figures. There was no invasion of the brain parenchyma. [file 13058_2023_1679_MOESM4_ESM.tif]

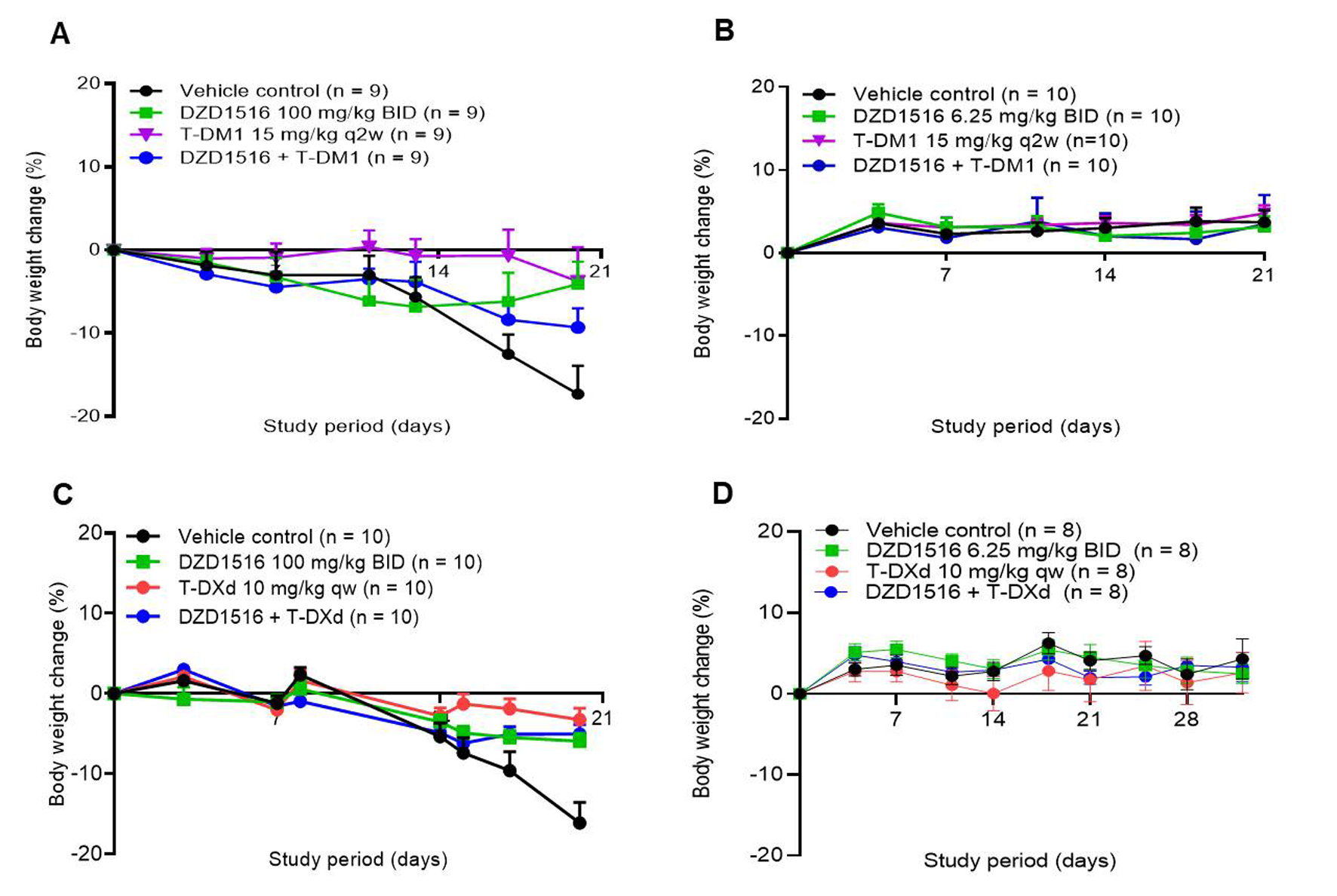

Supplement: Supplementary file 5 — Additional file 5. Figure S3: Body weight change of DZD1516 in combination with T-DM1 or T-Dxd in BT474C1-Luci Mono1 xenograft models. A. Body weight change of DZD1516 in combination with T-DM1 in the BM xenograft mice model. n = 9/group. B. Body weight change of DZD1516 in combination with T-DM1 in the SC xenograft mice model. n = 10/group. C. Body weight change of DZD1516 in combination with T-DXd in the BM xenograft mice model. D. Body weight change of DZD1516 in combination with T-DXd in the SC xenograft mice model. BID: twice daily; q2w: once every two weeks. qw: once weekly. BM: brain metastasis. SC: subcutaneous. [file 13058_2023_1679_MOESM5_ESM.tif]

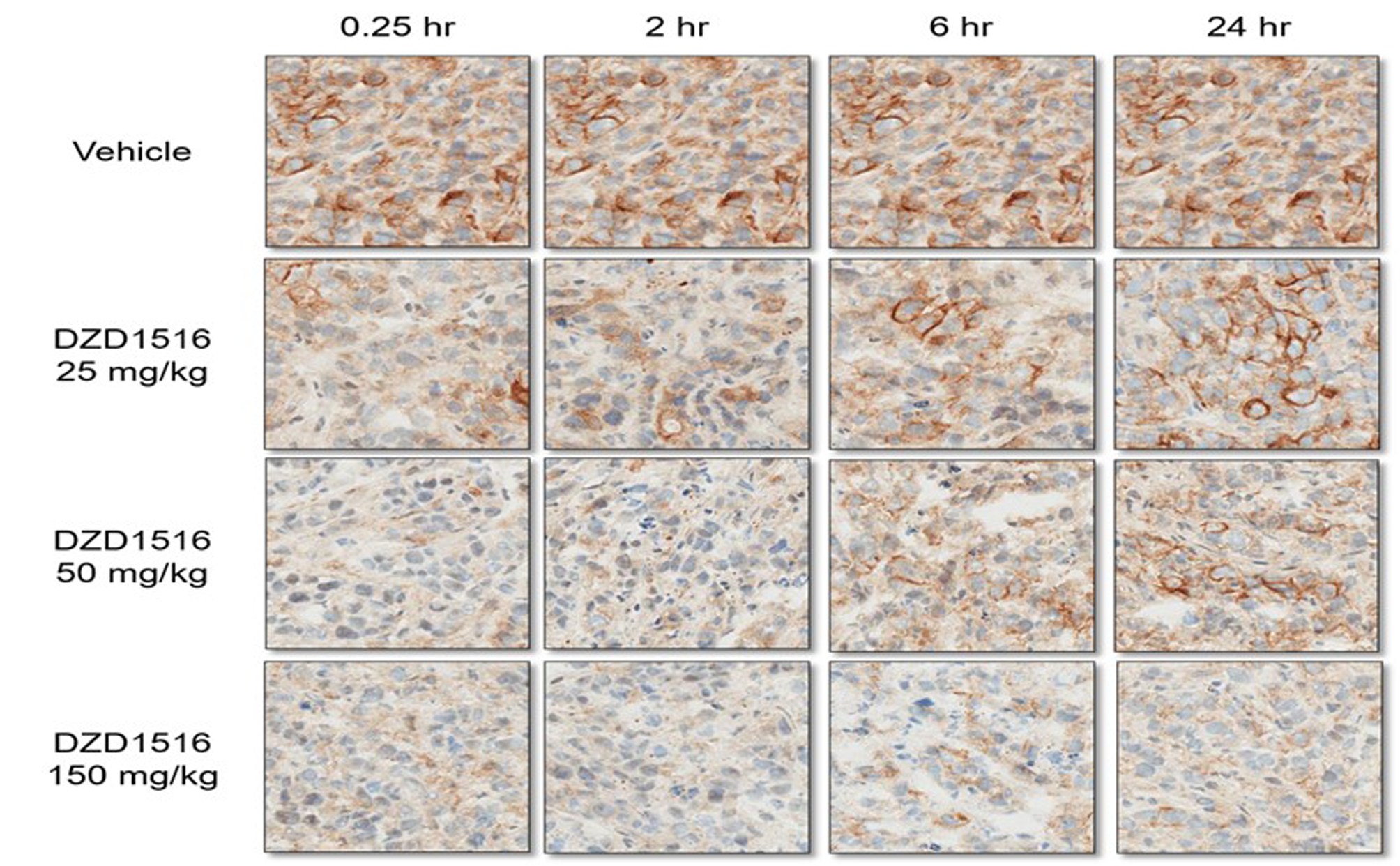

Supplement: Supplementary file 6 — Additional file 6. Figure S4: Representative images of pHER2 IHC post-single-dose of DZD1516 in subcutaneous BT474C1-Luci Mono1 xenograft model. The mice were treated with a single dose of DZD1516 at 25 mg/kg, 50 mg/kg, and 150 mg/kg, respectively. The tumor tissues at different timepoints post-single-dose of DZD1516 were collected, and pHER2 expression in the tumor tissues was analyzed by IHC assay. Each time point had tumor tissues from three mice to detect the pHER2 signal. IHC: immunohistochemistry. [file 13058_2023_1679_MOESM6_ESM.tif]

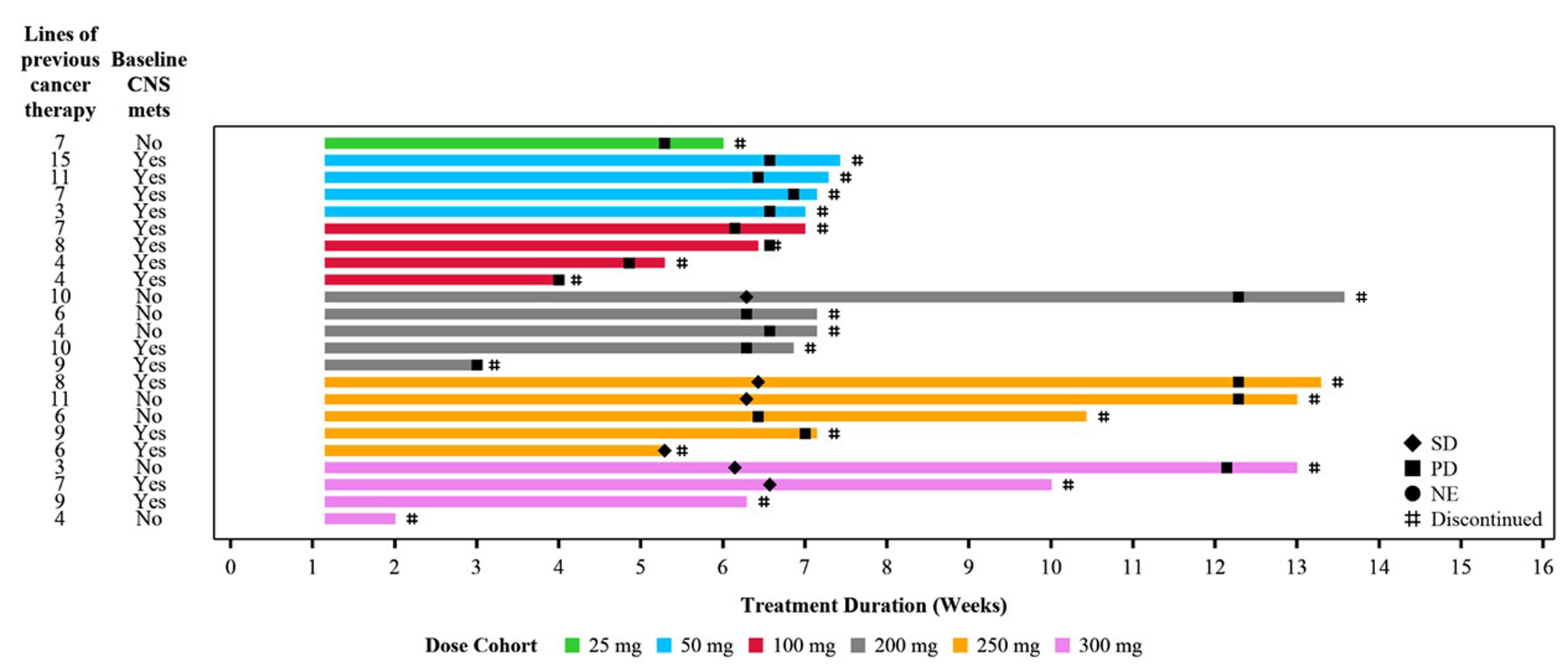

Supplement: Supplementary file 7 — Additional file 7. Figure S5: Clinical activity of DZD1516 in heavily pre-treated breast cancer patients. Swimmer plot of tumor response over time. The study drug was administrated since C0D1. Treatment duration was presented since C1D1. [file 13058_2023_1679_MOESM7_ESM.tif]
